# Genetic regulation of stem elongation and thickening in ornamental plants: a review

**DOI:** 10.1093/hr/uhag158

**Published:** 2026-04-17

**Authors:** Xi Chen, Chang Guo, Xingle Li, Meiting Wang, Xiaonan Yu, Wei Zhu

**Affiliations:** School of Landscape Architecture, Beijing Forestry University, Beijing 100083, China; Beijing Key Laboratory of Ornamental Plants Germplasm Innovation & Molecular Breeding, National Engineering Research Center for Floriculture, Beijing 100083, China; School of Landscape Architecture, Beijing Forestry University, Beijing 100083, China; Beijing Key Laboratory of Ornamental Plants Germplasm Innovation & Molecular Breeding, National Engineering Research Center for Floriculture, Beijing 100083, China; School of Landscape Architecture, Beijing Forestry University, Beijing 100083, China; Beijing Key Laboratory of Ornamental Plants Germplasm Innovation & Molecular Breeding, National Engineering Research Center for Floriculture, Beijing 100083, China; School of Landscape Architecture, Beijing Forestry University, Beijing 100083, China; Beijing Key Laboratory of Ornamental Plants Germplasm Innovation & Molecular Breeding, National Engineering Research Center for Floriculture, Beijing 100083, China; School of Landscape Architecture, Beijing Forestry University, Beijing 100083, China; Beijing Key Laboratory of Ornamental Plants Germplasm Innovation & Molecular Breeding, National Engineering Research Center for Floriculture, Beijing 100083, China; School of Landscape Architecture, Beijing Forestry University, Beijing 100083, China; Beijing Key Laboratory of Ornamental Plants Germplasm Innovation & Molecular Breeding, National Engineering Research Center for Floriculture, Beijing 100083, China; Environmental Horticulture Research Institute, Guangdong Academy of Agricultural Sciences, Guangzhou 510640, China

## Abstract

The stem, a crucial organ that connects the root and aboveground parts, is responsible for transporting water and nutrients. This review synthesizes the current understanding of stem development in ornamental plants. We first outline the morphological and physiological characteristics of stem elongation and stem thickening. Subsequently, we examine the roles of key genes, plant hormones, and cell wall components in regulating stem growth, mechanical strength, and overall plant architecture. We also analyze how environmental factors (e.g. temperature, light, water, and nutrients) and hormonal and genetic networks modulate stem development. Particular emphasis is placed on the functions of auxin, gibberellins, and brassinosteroids. Recent studies in ornamental plants such as *Prunus*, *Chrysanthemum*, and *Paeonia* have illuminated the advances in cultivation techniques and gene identification associated with cellular processes, cell wall synthesis, hormone biosynthesis, and signal transduction. Looking forward, we highlight emerging research directions, including the use of advanced imaging and artificial intelligence for phenotypic analysis, and the integration of multi-omics data within a ‘Breeding 5.0’ framework. Ultimately, this review aims to support the targeted breeding of ornamental plants with optimized stem traits, enhancing both aesthetic value and production efficiency.

## Introduction

Shoot architecture critically determines the morphology of the aboveground of ornamental plants, supporting their growth and ornamental value [1]. As an important aspect of shoot architecture, stem development plays a key role in transporting nutrients, water, and inorganic salts, and providing mechanical strength for plant growth. It involves two dimensions, longitudinal elongation and lateral thickening [2]. Stem elongation controls plant height, resulting in leaves capturing sunlight and the spread of pollen or seeds. An appropriate plant height is crucial for biomass accumulation, reproductive activities, and adaptability to different environments [3]. Cultivars with shorter stems generally exhibit higher yields and are economically beneficial for commercial value [4]. Stem thickness is closely related to the component alternation and anatomical characteristics. It gives rise to stem elasticity and rigidity, which determine the mechanical strength. In production, stem thickness is used as an important indicator for evaluating a plant’s ability to resist lodging. Therefore, stem development is fundamentally important for plant growth and productivity [5, 6]. Recently, research on the stem development of ornamental plants has gradually increased, involving temperature regulation, hormone signaling, and molecular mechanism elucidation, which provides an important theoretical basis and technical support for cultivation. In this review, we focus on the genetic regulatory mechanisms of stem development in ornamental plants, aiming to explore how new genes and mechanisms process stem architecture, as well as to propose current research hotspots and future directions.

## Biological basis of stem development

The stem is an important nutritional organ of the aboveground parts of plants. It can transport nutrients and water between roots and leaves, and function in supporting organs such as leaves, flowers, and fruits [7, 8]. The location where leaves are attached to the stem is called a node, and the part between two nodes is called an internode [9, 10]. Stem development encompasses two parts: stem elongation and stem thickening. Appropriate plant height and mechanical strength of the stem are crucial to upright plant growth, enhance plant resistance to lodging, increase plant biomass, and ultimately increase production [11–13]. This stem development is functionally integrated with root architecture. Healthy roots supply water, nitrogen, and nutrients necessary for sustained shoot growth [14, 15]. The plasticity of root traits is closely linked to the stability of shoot characteristics [16]. In turn, shoot architecture also influences root resource acquisition through carbon allocation priorities, and stem elongation often correlates with deeper root penetration to enhance lodging resistance [17]. In ornamental plant production, modification of stem development can cultivate dwarf potted varieties to increase their ornamental value [18, 19]. Therefore, the regulation of stem development has attracted increased attention and has been extensively studied.

### Morphological and physiological characteristics of stem elongation

**Figure 1:**
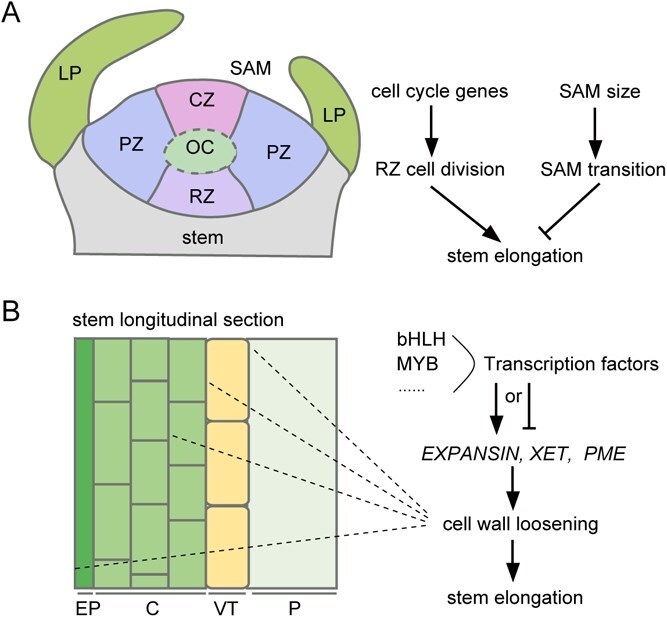
Genetic regulation of stem elongation. Stem elongation is determined by (A) early cellular activities in the shoot apical meristem (SAM) and (B) subsequent internode cell expansion. (A) SAM consists of central zone (CZ), organizing center (OC), and rib zone (RZ). Cellular activity in the RZ contributes to stem elongation, where cell division driven by cell cycle genes promotes elongation. An enlarged SAM accelerates the transition from vegetative to reproductive growth, which negatively impacts stem elongation. (B) Internode elongation depends on cell wall loosening. Transcription factors such as bHLH and MYB regulate the expression of *EXPANSIN*, *XYLOGLUCAN ENDOTRANSGLUCOSYLASE* (*XET*), and *PECTIN METHYLESTERASE* (*PME*), thereby modulating the degree of cell wall loosening and subsequently influencing cell and stem elongation. Note: EP, epidermis; C, cortex; VT, vascular tissue; P, pith. Arrows indicate positive regulation or promotion, while lines ending with vertical bars represent repression.

Stem elongation involves cell division (increasing the number of cells) and cell elongation (changing the cell size). The early stem elongation process is determined by the activity of the plant shoot apical meristem (SAM; shoot tip) [20, 21]. The SAM consists of the central zone (CZ), the organizing center (OC), and the rib zone (RZ) located below the CZ [22]. Among them, stem cell activity and cell division in the RZ region play important roles in stem elongation [23]. In the Arabidopsis SAM, the downregulated expression of cell cycle genes inhibits the division of stem elongation-related cells in the RZ [24] (Fig. 1A). The shape and size of the SAM also affect stem development. A larger SAM volume leads to early flowering and hinders stem elongation [25] (Fig. 1A). *FLOWERING LOCUS T* (*FT*) and *TERMINAL FLOWER 1* (*TFL1*) are two key genes involved in the SAM transition from vegetative growth to reproductive growth in plants. A high expression ratio of *FT/TFL1* promotes more vegetative meristems into inflorescence meristems, causing dwarf plant architecture [26].

During vegetative growth, plant height is determined by internode length (precisely cell length) and node number [27–29]. Histological observations of the internodes of dwarf mutants such as *semidwarf-1* (*sd1*) and *brassinosteroid insensitive 1* (*bri1*) show that the cell length is significantly reduced [30, 31]. The premise of internode cell elongation is loosening of the cell wall. The normal assembly of the cell wall can be influenced by the synthesis of cellulose, lignin, and other components (Fig. 1B). For example, mutations in genes encoding lignin synthases cinnamoyl-CoA reductase and cinnamyl alcohol dehydrogenase result in a severe dwarf phenotype [32, 33]. The expression of the transcription factor *MYB61* and cellulose synthase (*CesAs*) promotes cellulose biosynthesis, enhancing internode cell elongation [34, 35]. Additionally, cell elongation can be boosted by activating the expression of genes encoding *xyloglucan endotransglycosylase* (*XET*) and *expansin* (*EXP*) proteins, which cleaves xyloglucan polymers and destroy polysaccharide adhesion, thereby enhancing the loosening of the cell wall [36–38] (Fig. 1B). Multiple basic helix–loop–helix (bHLH) transcription factors, including ILI1 BINDING bHLH PROTEIN1, PACLOBUTRAZOL-RESISTANT1, and HOMOLOG OF BEE2 INTERACTING WITH IBH1, are jointly involved in the transcription of cell wall loosening–related genes, contributing to the elongation and growth of internode cells [39–41].

In some monocots, the primary meristem left over from the SAM is retained at the base of the internodes, namely, the intercalary meristem (IM). During the reproductive growth period, plants can undergo intercalary growth through the activity of the IM, causing dramatic stem elongation [42, 43]. Cell division, cell elongation, cell wall composition, and cytoskeleton activities in the IM collectively affect node number and internode length. In maize, both the *TERMINAL EAR 1* and *BELL1-like homeobox 12/14* genes are highly expressed in the IM. Their loss-of-function mutants present a dwarf phenotype with disordered IM development [44, 45]. Although multiple genes that regulate the homeostasis maintenance of the IM have been identified, the formation of the IM in ornamental plants and the detailed underlying mechanisms are still unclear.

### Morphological and physiological characteristics of stem thickness

**Figure 2:**
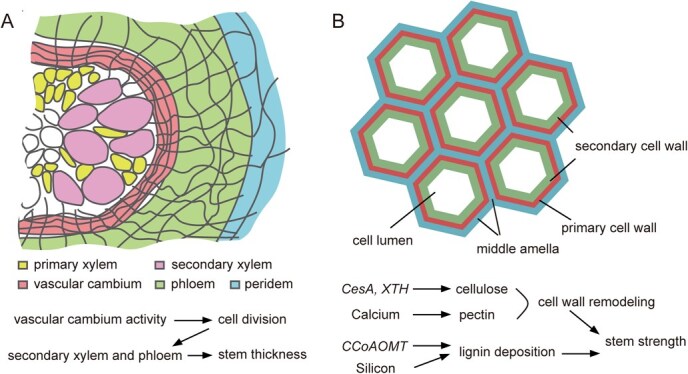
Cellular and molecular mechanisms underlying stem thickness. (A) Cross-section of stem showing secondary growth driven by vascular cambium activity. Cell division in the vascular cambium produces secondary xylem and secondary phloem, leading to increased stem thickness. (B) Structural components of plant cell wall contribute to mechanical strength and thickness. *CELLULOSE SYNTHASE* (*CesA*) and *XYLOGLUCAN ENDOTRANSGLUCOSYLASE/HYDROLASE* (*XTH*) promote cellulose synthesis; Ca^2+^ binds to pectin and supports cell wall integrity; silicon and *3-O-METHYLTRANSFERASE* (*CCoAOMT*) positively regulate lignin deposition into the cell wall. Together, these components enhance the structural integrity and mechanical strength of the stem.

Stem thickness is primarily determined by secondary growth, a developmental process driven by the activity of vascular and cork cambium (Fig. 2). This process produces secondary vascular tissues (secondary xylem and secondary phloem) and the periderm through vascular cambium and cork cambium activity [46, 47]. Through cell division, differentiation, and secondary cell wall deposition, this process increases stem diameter and confers significant mechanical strength (Fig. 2A). Stem strength is the key agronomic trait for plants to resist pests and lodging and is influenced by the number and arrangement of vascular bundles, secondary cell wall thickness, and lignin content [13, 48].

The synthesis of secondary cell walls is crucial to mechanical strength (Fig. 2B). Moreover, the cell wall provides the elasticity required for cell expansion by stretching itself, absorbs new polysaccharide polymers to maintain its thickness and toughness, and provides rigid support for plant tissues and organs [49,50]. The cell wall consists of cellulose, hemicellulose, pectin, protein, and mineral elements such as calcium and silicon. Cellulose is the main component of the cell wall from the cellulose synthase complex on the plasma membrane [51]. The *CesA* family plays an important role in the synthesis of both primary and secondary cell walls [52] (Fig. 2B). Hemicellulose or pectin side chains in cellulose microfibrils can change the properties of cellulose and reduce its mechanical strength, ultimately causing the bending of plant stems. In a study on six kinds of cut flowers, including lotus, water lily, rose, gerbera, lisianthus, and carnation, the existence of hemicellulose or pectin side chains in curved stems, not upright stems, disrupted the crystallinity, purity, and length of cellulose, resulting in the acceleration of cell wall degradation and decreased mechanical strength [53, 54].

In the comparative analysis of large and small lotus stems, the differentially expressed xyloglucan endotransglucosylase/hydrolase genes (*XTH*) are identified as an important gene family involved in cell wall remodeling in lotus architecture formation (Fig. 2B). The overexpression of *XTHs* blocks the gravity response during the development of the secondary cell wall, decreasing the stem’s mechanical strength [55, 56]. Lignin can enhance the rigidity and compressive strength of cell walls by filling the interstitial spaces between cellulose microfibrils. Many studies have focused on how to regulate the mechanical strength of ornamental plant stems by altering the lignin content. In herbaceous peony, there is a significant positive correlation between mechanical strength and lignin content [57, 58]. In addition, the lignin synthase 3-O-methyltransferase (CCoAOMT) gene is highly expressed in xylem tissues in *Forsythia*. The overexpression of *FsCCoAOMT* enhances the stem mechanical strength [59] (Fig. 2B).

Calcium is an indispensable component of the cell wall, where Ca^2+^ can bind with the free carboxyl groups in pectin to form stable complexes, thereby improving the rigidity and stability of cell walls [60] (Fig. 2B). Ca^2+^ reduces the ethylene level produced by gravity and significantly inhibits bending in *Antirrhinum majus* [61]. In gerbera and herbaceous peony, exogenous Ca^2+^ treatments improve stem quality by increasing stem strength and reducing bending [62–64]. Silicon is present in the tissues of nearly all terrestrial plants and is primarily deposited as amorphous silica in specific cell walls [65] (Fig. 2B). It plays a key role in the assembly and remodeling of cell walls [66]. In herbaceous peony, silicon application enhances stem strength by promoting lignin accumulation [67].

Furthermore, a reasonable stem structure enhances plant adaptability to abiotic stresses. Specifically, stem secondary development enhances drought tolerance by modulating vessel density and lignin deposition to optimize water transport efficiency [68]. Specialized stem porous structure enhances wind resistance by effectively dissipating the load [69, 70]. Additionally, the specific xylem secondary growth patterns regulate ion transport, which helps maintain ionic homeostasis and improves salt tolerance [71]. Overall, stem secondary development not only underlies morphological maturation but also provides an integrated structural foundation for adaptation to biotic and abiotic challenges (Fig. 2).

## Effects of hormones on the development of ornamental plant stems

Plant hormones are crucial regulators of stem development in ornamental plants. Among them, auxin, gibberellins (GAs), and brassinosteroids (BRs) are three important plant growth–promoting hormones that significantly control plant height by regulating stem cell division, cell elongation, and tissue differentiation. In horticultural production, chemical plant growth regulators (PGRs) are commonly employed to control plant height, as detailed in Table 1.

**Table 1:** Hormone treatments regulate the stem development in ornamental plants.

**Treatment**	**Concentration**	**Species**	**Effect** ^**a**^	**Mechanism**	**Reference**
	0.02, 0.04, 0.06, 0.08, and 0.1 mg·l^−1^ BL	*Matthiola incana*	Positive	0.06 and 0.08 mg·l^−1^ BL promoted plant height, stem diameter, fresh weight, and chlorophyll content.	[72]
Brassinolide (BL)	0.05 mmol·l^−1^2,4-epibrassinolide (EBR)	*Lilium* hybrid	Positive	BL spraying promotes plant height and dry weight	[73]
	0.67 mM (6-BA) 6-benzyladenine	*Paulownia* ssp.	Positive	6-BA stimulated stem elongation and induced a transient stem thickening 1 week after application.	[74]
	0.002 mg·l^−1^ Thidiazuron (TDZ)	*Rhododendron aureum*	Positive	The combination of 0.002 mg·l^−1^ TDZ + 0.5 mg·l^−1^ IBA was optimal for stem elongation *in vitro*.	[75]
Cytokinin	10, 20, 40, 80, and 100 μM TDZ	*Rosa hybrida*	Dual-effect	100 μM TDZ reduced new shoot length to half that of the control while increasing stem diameter by ~40%.	[76]
	150 mg·l^−1^ GA_3_	*Anemone* spp.	Positive	GA_3_ significantly affected stem height, number of leaves, and flower behavior.	[77]
	50, 100, and 150 mg·l^−1^ GA_3_	*Cyclamen africanum*	Positive	150 mg·l^−1^ GA_3_ significantly increased plant height and petiole length.	[78]
	100 and 200 mg·l^−1^ GA_3_	*Phalaenopsis* spp.	Dual effect	GA_3_ increased stem length and decreased stem diameter.	[79]
	10, 25, and 50 ml Promalin (50 mg·l^−1^ each GA_4 + 7_ and BA)	*Lilium longiflorum*	Positive	Root-absorbed GA_4+7_ (25 and 50 ml) promoted stem elongation, with spray volumes >10 ml per plant increasing over-elongation risk.	[80]
Gibberellin	GA_4 + 7_, GA_3_, PBZ (1, 10 and 100·l^−1^)	*Cyclamen persicum*	Positive	GAs could always increase plant height under different photoperiods and temperatures.	[81]
	0.25, 0.5, 1, or 2 mg·l^−1^ Paclobutrazol (PBZ)	*Iris nigricans*	Negative	PBZ at 0.5 or 1 mg·L^−1^ caused severe dwarfism, characterized by drastic reductions in stalk height and weight.	[82]
	400 mg·l-1 PBZ	*Agapanthus praecox*	Negative	400 mg·l^−1^ PBZ shorten the scape length over 70%.	[83]
	100, 300, and 500 mg·l-1 PBZ	*Lilium longiflorum*	Negative	Plants are more severely suppressed and become shorter as PBZ concentration increases.	[84]
	30 mg·l^−1^ PBZ	*Cyclamen* sp.	Negative	PBZ produced a smaller plant height and peduncle length.	[85]
Gibberellin inhibitor	50, 150, and 300 mg·l^−1^ Uniconazole (UCZ)	*Curcuma alismatifolia*	Dual-effect	UCZ-treated plants exhibited obviously decreased plant height and scape length, while stem diameter increased slightly.	[86]
Melatonin	0.5 mM Melatonin once a week	*Paeonia lactiflora*	Positive	Melatonin treatment enhanced stem strength, lignin content, and secondary cell wall thickness.	[87]
Methyl jasmonate	200 mg·l^−1^ MeJA	*Salix purpurea*	Positive	MeJA significantly increased plant height, shrub diameter, and branch diameter.	[88]
Flurprimidol	0.02, 0.04, 0.08, 0.16, and 0.24 mg per pot	*Lilium longiflorum*	Negative	Flurprimidol drenches suppressed lily height by up to 59% in a dose-dependent manner, with no effect on flowering.	[89]

a Effect categories: positive = promotes the stem developmental process(es) (elongation and/or thickening); negative = suppresses the stem developmental process(es) (elongation and/or thickening); dual-effect exerts opposing actions on elongation and thickening. Control plants were treated with distilled water.

### Auxin is crucial for stem development

**Figure 3:**
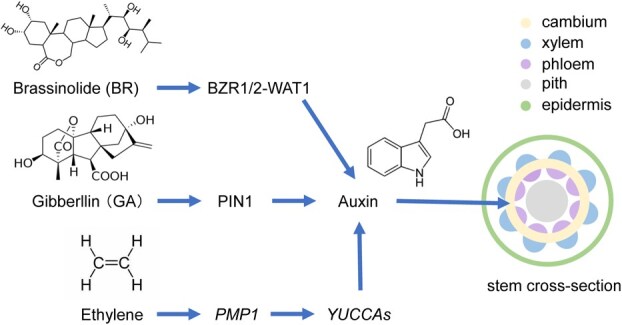
Plant hormones crosstalk coregulates the differentiation of cambium stem cells into xylem. Brassinosteroid (BR) signaling, acting through the BRASSINAZOLE RESISTANT1/2-WALLS ARE THIN1 (BZR1/2-WAT1) module, promotes local auxin accumulation. Gibberellin (GA) upregulates the auxin transporter PIN-FORMED 1 (PIN1) to facilitate auxin polar transportation, while ethylene induces *PETAL MOVEMENT-RELATED PROTEIN 1* (*PMP1*), which activates *YUCCA* genes to stimulate auxin biosynthesis. Consequently, auxin functions as a central signal to direct cambial cell fate toward xylem differentiation. Note: Stem cross-section illustrates the spatial organization of distinct tissue regions. Each color corresponds to a specific tissue type in the legend.

Auxin plays a central role in promoting cell division, extension, and differentiation during plant growth [90, 91]. Through an elaborate network of biosynthesis, polar transport, and signal transduction, auxin establishes a tissue-specific concentration distribution pattern, regulating stem elongation and secondary growth (Table 1; Fig. 3).

Auxin biosynthesis mainly takes place in the SAM and young leaves. Exogenous application of Indole-3-acetic acid (IAA) can significantly promote flower stem elongation [92]. Subsequently, auxin is polarly transported to specific tissues via PIN-FORMED (PIN) proteins, resulting in well-defined spatial distribution patterns [93], and accurately coordinate the differentiation ratio of xylem and phloem in the cambium [94, 95] (Fig. 3). The physiological functions of auxin are ultimately executed through its signal transduction pathway, achieving precise control over downstream target gene expression. The classical signaling pathway includes auxin receptors (TRANSPORT INHIBITOR RESPONSE 1/AUXIN SIGNALING F-BOX PROTEIN, TIR1/AFB), Aux/IAA transcriptional repressors, and AUXIN RESPONSE FACTOR (ARF) proteins [96]. Mutations in IAA genes can lead to dwarf phenotypes in *Brassica napus* [97, 98] (Table 1).

### Gibberellins regulate stem internode elongation

**Figure 4:**
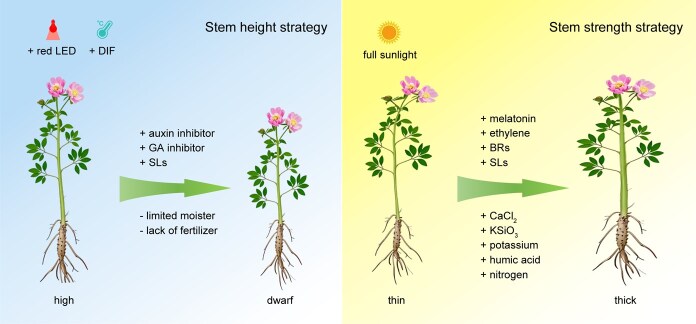
Strategies for regulating plant architecture of ornamental plants. Two key horticultural approaches are illustrated: stem height control (left side) and stem strength enhancement (right side). For stem height control, dwarfing can be achieved under red LED light, positive day and night temperature (DIF) conditions, exogenous plant growth regulators (auxin inhibitors, GA inhibitors, and SLs), and environmental factors (limited moisture and fertilizer deficiency). For the stem strength strategy, stem thickening can be enhanced under full sunlight, exogenous plant growth regulators (melatonin, ethylene, BRs, and SLs), and nutritional supplements (e.g. CaCl₂, KSiO₃, potassium, humic acid, and nitrogen). Note: The ‘+’ symbol indicates the presence of treatments. The ‘−’ symbol indicates the absence of treatments or limited condition.

GAs are widely involved in all stages of stem development of ornamental plants. They are mainly synthesized in SAM, root meristem, seed, and fruit [99]. The application of GA synthesis inhibitors such as paclobutrazol has been demonstrated to effectively induce dwarfism in pot flowers and increase their ornamental value [84] (Table 1; Fig. 4). The precursor substance is geranylgeranyl pyrophosphate (GGPP), which is converted into active gibberellins GA_1_, GA_3_, GA_4,_ and GA_7_ through a series of enzymatic reactions [100]. GAs promote cell elongation and expansion by mediating cell wall elasticity and regulate the xylem/phloem ratio in the vascular cambium [94].

In Arabidopsis, mutations in early GA synthesis-related genes (*ent-copalyl diphosphate synthase*, *CPS*; *ent-kaurene synthase*, *KS*; *ent-kaurene oxidase*, *KO*; *ent-kaurenoic acid oxidase*, *KAO*) result in a severe dwarf phenotype, whereas mutations of later GA synthesis–related genes (*GA20oxidases*, *GA20ox*; *GA3oxidases*, *GA3ox*) result in a semidwarf phonotype, and dwarf defects can be restored by exogenous treatment of GAs [101–103]. In *Chrysanthemum*, GA_4_ accumulation is high in elongating internodes, and B-type GA receptor genes are abundantly expressed in both the internodes and leaves of extending shoots [104]. Knock-down mutants of *CmGA20ox* [105] and *CmGA3ox* [106] can form dwarf chrysanthemum varieties. *GA2 oxidases* (*GA2ox*) are involved in the catabolism of GA to maintain homeostasis. Comparative transcriptome analysis of dwarf and normal varieties revealed that the overexpression of *GA2ox* caused a semi-dwarf phenotype in chrysanthemum [107] and *Prunus* species [108].

In GA signaling, GAs can relieve the inhibition effect of DELLA proteins on plant growth through the GA receptor GIBBERELLIN-INSENSITIVE DWARF1 (GID1) [103, 109]. In *Arabidopsis*, *gid1* mutants show shorter stems [110]. Moreover, DELLA proteins are unable to perceive GA signals, resulting in short stems, dark green leaves, and late flowering [111]. The heterologous overexpression of the Arabidopsis *GIBBERELLIN INSENSITIVE* (*GAI*) gene in chrysanthemum can be used to cultivate dwarf lines [112]. In the future, manipulating GAs and their inhibitors in the cultivation of ornamental plants can effectively control plant height, breed novel varieties, and improve ornamental value.

### Brassinosteroids participate in cell wall remodeling and promote stem elongation

BRs are a type of sterol hormone widely found in plants that regulate important agronomic traits such as leaf angle and plant height. In the early stage of cell elongation, the cell wall loosening genes *XTH*s and *EXP*s are induced by BRs and then promote cell wall remodeling and cell expansion during stem growth [113, 114]. Moreover, BRs induce the synthesis of the receptor-like protein kinases HERCULES receptor kinase 1 and THESEUS1, which are required for the expression of *XTHs* and *EXPs* in the cell wall remodeling process and stem elongation [115].

Dwarf mutants that are defective in BRs biosynthesis or signaling pathways exhibit hypocotyl shortening and reduced plant height, while exogenous BR treatment can restore abnormal phenotypes, indicating that BRs play a key role in stem development [116, 117] (Table 1; Fig. 4). The key transcription factor BRI1 EMS SUPPRESSOR1/BRASSINAZOLE RESISTANT1 (BES1/BZR1) in the BR signaling pathway, directly regulates the expression of cell cycle–related genes, promoting cell division and stem elongation [118] (Fig. 3). In the study of ornamental plants, the analysis of differentially expressed gene in the dwarf plants *Dendranthema morifolium* and *Agapanthus praecox* revealed that the BR content and the expression of BR-related genes were reduced, suggesting that dwarf cultivars can be bred by regulating BR content and signaling [83, 105] (Table 1).

### Hormone signaling crosstalk synergistic regulation of stem development

In plants, the continuous division and differentiation of the vascular cambium during stem development are precisely coordinated by multiple plant hormone signaling pathways. Among them, auxin not only maintains the activity of cambium stem cells but also serves as a core signaling hub, establishing extensive crosstalk with other key hormones such as GAs, BRs, and ethylene, jointly regulating the fate determination and tissue differentiation of the cambium [119] (Fig. 3).

The integration among these hormone pathways often depends on specific transcriptional regulatory modules. In *Arabidopsis thaliana*, the BRASSINAZOLE RESISTANT-AUXIN RESPONSE FACTOR-PHYTOCHROME INTERACTING FACTORS (BZR–ARF–PIF) module regulates cell elongation genes to control hypocotyl elongation, with DELLA proteins acting as repressors of this module [120, 121]. The BRASSINAZOLE RESISTANT1/2-WALLS ARE THIN1 (BZR1/2-WAT1) module promotes xylem differentiation in the vascular cambium by increasing local auxin signaling level [122]. In addition, BRs synergistically interact with GAs and auxin coregulates stem development [123–125].

GAs can reinforce auxin signaling, promoting cambium homeostasis. ARF7 mediates interactions between DELLA and auxin signaling ARF/IAA proteins to regulate cambial activity [126]. GAs promote *PIN1* expression in the vascular tissues, causing cambium stem cells to preferentially differentiate into xylem cells [94]. This hormonal synergy is particularly evident during wound-induced regeneration; in grafting and peeling processes, the auxin signal integrates with GAs to promote the regeneration of vascular cambium [127, 128].

Notably, this multi-hormone integration mechanism also operates in ornamental plants. In rose, ethylene-induced *PETAL MOVEMENT-RELATED PROTEIN 1* (*RhPMP1*) upregulates the auxin synthesis genes *RhYUCCA3* and *RhYUCCA4* to maintain the activity of stem cells in the cambium [129]. This further illustrates how distinct hormonal pathways effect on local auxin distribution pattern to regulate stem development.

### Other hormones participate in the regulation of stem development

In addition to auxin, BRs, and GAs, other plant hormones such as cytokinins (CTKs), strigolactones (SLs), and ethylene are involved in regulating the development of plant stems. Many studies have revealed that exogenous hormone treatment efficiently improves the stem phenotype of ornamental plants.

CTKs affect stem growth mainly by regulating cell division and elongation. In *Arabidopsis*, Cytokinin B response factors can directly activate the expression of the stem cell maintenance genes *WUSCHEL* and *CLAVATA*, enhancing the activity of the SAM [130, 131]. Moreover, CTK treatment promotes cell division by regulating the expression of the cell division cycle–related genes *CYCLINS*, laying a foundation for stem development [132]. In switchgrass, the increase in CTK and GAs can promote cell elongation and vascular bundle development, thereby increasing plant height [133].

SLs play dual regulatory roles in stem development by modulating both primary elongation and secondary growth (Table 1). During early stem elongation, SLs specifically inhibit hypocotyl elongation by promoting the accumulation of the ELONGATED HYPOCOTYL 5 (HY5) protein [134]. In contrast, during secondary growth, SLs convert long-distance auxin signals from the shoot apex into cellular division and differentiation responses, directly promoting cambial cell division and the proliferation of xylem and phloem, thereby driving stem thickening [135]. In addition, SLs negatively regulate shoot gravitropism, change the stem angle, and subsequently alter plant photosynthetic efficiency [136].

In dicots, ethylene stimulation leads to a triple response: inhibiting stem elongation, hypocotyl swelling, and apical hook exaggeration (Fig. 4). In stem development, ethylene controls the activity of vascular cambium stem cells by promoting auxin biosynthesis. In woody rose, ethylene affects auxin synthesis *RhYUCCA10* and auxin transporter protein RhAUX2 by regulating *RhPMP1*, promotes stem cambium activity, and accumulates auxin in the cambium near the xylem [129]. The ethylene response factors ERF11 and ERF109 integrate auxin and gibberellin biosynthesis pathways to suppress internode elongation. In addition, ethylene can induce microtubule reconstruction to enhance hypocotyl elongation [137, 138]. In contrast, ethylene promotes stem elongation under water stress. Flooding causes an increased accumulation of ethylene in the stems, which promotes the synthesis and signal transduction of GAs and rapid stem elongation to resist stress. These studies show that ethylene has a dual effect on stem elongation [139–141].

## Effects of environmental factors on stem development

### Optimizing temperature management to regulate internode elongation in ornamental plants

Temperature is one of the key environmental factors affecting the development of ornamental plant stems [142] (Table 2). The temperature-dependent growth responses are not merely physiological adjustments but are based on conserved thermosensing mechanisms. In *Arabidopsis*, four major thermosensors, Early Flowering 3 (ELF3), UV Resistance Locus 8 (UVR8), cryptochrome 2 (CRY2), and Phytochrome B (PhyB), work synergistically to enable plants to perceive temperature changes [143]. The thermosensors activate downstream signaling cascades involving key transcription factors such as PHYTOCHROME-INTERACTING FACTOR 4 (PIF4), which integrates hormonal pathways, including auxin, GAs, ethylene, and BRs, to promote cell elongation and modulate stem growth [144].

**Table 2:** Effects of environmental factors on stem development.

**Factor**	**Treatment**	**Control**	**Species**	**Effect**	**Mechanism**	**Reference**
	Drought-stressed plant	Well-watered plant	*Hibiscus acetosella*	Stem elongation	Limited moisture reduced stem elongation by 44% and final plant height by 21% compared to well-watered conditions.	[145]
Moisture	50% of the control, 25% of the control	—	*Callistemon citrinus*	Stem elongation and thickening	Limited moisture led to a reduction of 12% of total biomass, reduce both stem diameter and plant height.	[146]
	Blue light: 16-h light/8-h dark	White light	*Chrysanthemum morflorium*	Stem thickening	After 60 days of blue light treatment, plants had thinner stems than controls.	[147]
	Green LED, Red LED, Blue LED, and Mix RGB light	White light	*Tulipa* sp.	Stem elongation	Green and blue light produced longer stems and internodes than red or RGB light. Red and RGB light produced the shortest stems at full bloom.	[148]
	20 μmol m^−2^ s^−1^ Red LED	White light	*Chrysanthemum morifolium*	Stem elongation	Red light resulted in shorter stems and poorer-quality branches than constant light.	[149]
	EOD-Far Red (FR) light	Ambient light	*Eustoma grandiflorum*	Stem elongation	EOD-FR enhanced stem elongation, specifically increasing the length of internode and pith cell.	[150]
	630 nm EOD Red light 730 nm EOD FR light	Cool white light	*Euphorbia pulcherrima*	Stem elongation	30 min EOD Red light resulted in a 34% ~ 54% reduction of shoot and internode length compared to EOD FR treatment.	[151]
	High-pressure sodium lamps with 600–700 nm red LED or 400–500 nm blue LED	High-pressure sodium lamps	*Petunia* sp.	Stem elongation	Blue light promoted stem elongation, caused upright shoot orientation; however, red light reduced shoot elongation	[152]
Light quality	700 ~ 800 nm FR light 40 μmol m^−2^ s^−1^	White light	*Antirrhinum majus Zinnia elegans*	Stem elongation	FR treatment increased the seedling height of *A. majus* by 64% ~ 134% and *Z. elegans* by 52% ~ 96% than controls.	[153]
	20% natural light	Natural light	*Chrysanthemum morifolium*	Stem elongation	Under 20% natural light treatment, the internode length increased. The epidermis and cortical and vascular bundle cells were deformed.	[154]
						
Light intensity	60% natural light	Natural light	*Paeonia lactiflora*	Stem elongation and thickening	Plants exposed to full sun exhibited greater plant height, stem diameter, branch number, and node number than those in shade.	[58]
	Four solutions NH_4_^+^-N∶NO_3_-N	NH_4_ + -N:NO_3_-N = 0∶100	*Lilium orential*	Stem elongation and thickening	NH_4_^+^-N∶NO_3_-*N* = 25∶75 (N_3_) treatment had the highest values in plant height and flower diameter	[155]
	1000 μl/l CO_2_+ 0.85 μl/l NO_x_ for 42 days	1000 μl/l CO_2_ for 42 days	*Rosa* sp.	Stem elongation	NO_x_ addition resulted in shorter stems in roses	[156]
	Purified phytase enzyme (0 EU、 5 EU、10 EU) soaking 15, 30, and 60 min	Distilled water	*Brassica oleracea*	Stem thickening	10 EU of purified phytase enzyme produced the highest stem diameter under 30 and 60 min treatments.	[157]
	140 g·m^−3^ KSiO_3_	Complete fertilizer without KSiO_3_	*Helianthus annuus*	Stem elongation and thickening	KSiO_3_ treatment plant showed thick, straight stems, increased flower and stem diameters, and increased height compared to the control group.	[158]
Fertilizer	Humic acid at 1:600 (v/v) concentration	0.3% NPK fertilizer (N:P_2_O_5_:K_2_O = 16:6:20)	*Chrysanthemum morifolium*	Stem thickening	Humic acid obviously improved stem diameter and shoot weight compared to the control and the NPK fertilizer.	[159]
Photoperiod	Different photoperiods 8, 10, 12, 14, 16, or 18 h	—	*Salvia exserta*	Stem elongation	A photoperiod over 8 h increased plant height by 22%–55% and 14 h of light increased node number.	[160]
Temperature	Six soaking temperatures and times in warm water	—	*Lilium orential*	Stem elongation	Bulbs treated with warm water (48°C for 3 min, repeated three times) and then in 45°C water for 30 min improved plant height.	[161]

In orchid production, the growth rate of plant stems can be regulated by adjusting the day temperature [162]. Low temperature can inhibit hypocotyl and stem elongation. For example, warm-water treatment of lily bulbs can promote their vegetative growth and significantly increase plant height [161]. In chrysanthemum, internode length is dependent on the difference between day and night temperature (DIF) [163]. By plotting the curves showing that internode elongation changes with temperature and establishing a prediction model and formula for internode length, it was found that DIF and the absolute temperature independently regulate internode length. When DIF is positive (daytime temperature is higher than the nighttime temperature), the chrysanthemum internode length increases. Conversely, a negative DIF inhibits internode elongation, causing plant dwarfing [164] (Table 2; Fig. 4). Recent studies have focused on the reasonable regulation of DIF and absolute temperature levels to accurately manage the stem growth of ornamental plants [165].

### Light intensity, light quality, and photoperiod intricately regulate stem development

Light plays an important role in plant growth. It provides energy for plant photosynthesis and acts as a signal to regulate plant photomorphogenesis, including cell differentiation, stem elongation, and secondary growth. Plants perceive light through specific photoreceptors: CRY sense blue light, phytochromes (Phy) detect red (R) and far-red (FR) light, and UVR8 perceives ultraviolet-B. These receptors initiate signal transduction involving key regulators such as transcription factors PIFs, E3 ubiquitin ligase CONSTITUTIVE PHOTOMORPHOGENIC 1 (COP1), and HY5, ultimately modulating downstream gene expression and physiological responses [166]. Appropriate light conditions (i.e. light quality, light intensity, and photoperiod) can promote leaf photosynthesis and provide sufficient nutrients and energy for stem growth [167–169] (Table 2).

To cope with light competition, plants can adjust their stem elongation to the growth of surrounding vegetation under low-light conditions, which is referred to as the shade avoidance reaction (SAR) [170]. Light signaling pathways are closely integrated with hormonal networks; for instance, under shade conditions, the synthesis and signaling of auxin, GA, SL, and ethylene are enhanced, collectively reshaping shoot architecture [171]. In sun-loving plants such as *B. napus*, the elongation of stem internodes and petioles is accelerated, the number of branches decreases, and flowering occurs earlier through SAR, while high light intensity reduces plant height [172, 173].

Different light wavelengths trigger distinct regulatory pathways in stem development. Blue light regulates plant phototropism, chloroplast migration, and leaf expansion [174]. In canola, irradiation with 400 ~ 500 nm blue light increases plant height but makes stems thinner [172]. Overexpression of the blue light receptor *CRYPTOCHROME 1 (CRY1)* inhibits cell elongation in the hypocotyl [175]. Green light participates in the photosynthesis process to promote leaf development and stem elongation while reducing plant biomass production [148, 176]. Moreover, red light is involved in photosynthetic organ development and assimilate transport. The overexpression of phytochrome B1 (PhyB1) in chrysanthemum can hinder plant vegetative growth and reduce plant height [177].

In addition to light quality, photoperiod also plays a key role in regulating stem growth and has significant implications for production costs. Cultivars with long photoperiods increase production costs, and a reasonable short-day treatment or the selection of short-photoperiodic varieties could help control costs. Chrysanthemum flowers are sensitive to both photoperiod and light quality [178]. Therefore, understanding the interaction between light quality and photoperiod is essential for optimizing plant architecture and improving economic efficiency in production.

### Water and fertilizer affect stem quality by influencing cell turgor pressure and mechanical strength, respectively

Water is essential for maintaining cell turgor pressure [179]. Adequate water promotes stem thickening and elongation and improves plant lodging resistance. Moreover, water is involved in various physiological processes such as photosynthesis and respiration, providing the necessary material and energy basis for stem growth (Table 2; Fig. 4) Insufficient water will decrease the turgor pressure of stem cells, making the stems thin and weak [180]. In *Penstemon* × ‘Ruby Candle’, the lack of water results in short internodes [181]. Conversely, excessive water supply in *Prunus species* leads to poor soil aeration, weak root functions, and plant diseases and then hinders stem development [182].

Fertilizers are important substances for maintaining plant growth and increasing productivity [183]. Adequate fertilization supplies nutrients for stem elongation and stem thickening, while overuse of fertilization is harmful to plants, soil, water, and the environment [184] poinsettia and chrysanthemum [185]. In addition, potassium (K) and silicon (Si) fertilizers increase stem thickness, enhance mechanical strength, and improve the photosynthetic efficiency of ornamental plants. High potassium treatment promotes photosynthesis, seedling growth, and flowering in chrysanthemum. In addition, the application of silicon fertilizer to petunia makes stem epidermal cells siliconized and enhances stem mechanical strength, causing straight stems and improved lodging resistance [186–188] (Table 2).

Therefore, in the ornamental plant cultivation process, adjusting water and fertilizer supplies according to the growth demand and environmental conditions results in a higher stem quality (Fig. 4).

## Multi-omics and genetic transformation studies of stem development in ornamental plants

### Integrated multi-omics analyses insights into stem development in ornamental plants

Stem development in ornamental plants is a complex biological process that is regulated by a combination of genetic and environmental factors. Recent advances in omics techniques (genomics, transcriptomics, metabolomics, and proteomics) have provided an effective approach for studying the underlying regulatory mechanisms [189–191].

Genome sequencing has laid a foundational resource for ornamental plant research, enabling comprehensive characterization of genome architecture, identifying genetic variation, elucidating phylogenetic evolution, and deciphering population structure. These insights further enable the prediction of population responses to future environmental changes [192] and lay the foundation for the identification of functional genes [193]. For example, whole-genome sequencing of multiple *Prunus* species has identified many quantitative trait loci (QTL), markers and genes such as *PmTAC1* linked to plant architecture, such as weeping [191, 194, 195] (Table 3).

**Table 3:** Application of multi-omics in studying stem development genetics of ornamental plants.

**Omic type**	**Species**	**Material**	**Main conclusion**	**Reference**
Genome	*Rosaceae*	Nine *Rosaceae* species	*MdABCG28*, a possible cytokinin transporter linked to the dwarfing phenotype in apple rootstocks.	[196]
	*Chrysanthemum*	200 chrysanthemum accessions	Genome-wide association studies identified 19 genes related with plant height development.	[1]
Transcriptome	*Gerbera hybrida*	3 developmental stages of stem	Analysis of Differentially expressed genes (DEGs) in hormone pathways suggested ABA regulates stem bending independently of ethylene.	[197]
	*Rhododendron canescens*	Vegetative and reproductive tissues	Identified plant hormone genes *GAI, GID1, BRI1, MAX2, BRC1,* etc., related to plant height and branching.	[198]
	*Liquidambar styraciflua Liquidambar. formosana*	Tetraploid and diploid hybrid sweetgum stem	DEGs were significantly enriched in plant hormone biosynthesis and signal transduction, sugar and starch metabolism, and cell cycles.	[199]
	*Ilex verticillate*	Long stem cultivar ‘Oosterwijk’ Short stem cultivar ‘Red sprite’	Candidate DEGs associated with stem length involve phenylpropanoid biosynthesis, phenylalanine metabolism, and the auxin signaling pathway.	[200]
	*Agapanthus praecox*	400 mg·L^−1^ PBZ treated scape Distilled water–treated scape	DEGs enriched in hormone signaling, carbohydrate metabolism, and cell wall–related biological processes.	[83]
	*Lagerstroemia* sp.	Dwarf and nondwarf progenies of an F1 segregating population	DEGs involved in phytohormone pathways and cellular patterning regulation.	[201]
	*Taxus mairei*	15 ~ 20 young stems were cut into 0.1 ~ 0.2 mm fragments	scRNA-seq has elucidated cell wall synthesis in stems by identifying distinct cell populations such as cambium, xylem, and phloem cells.	[202].
Genome and Transcriptome	*Prunus mume*	Young leaves for genome sequencing Straight and tortuous branches for transcriptome	Identified genes involved in cell division, development, and plant hormone signaling are essential for the tortuous branch trait formation.	[195]
Transcriptome and metabolome	*Nymphaea tetragona*	Dorsal and ventral stems were sampled on the fifth day after cutting	607 DEGs identified in the dorsal and ventral stems revealed significant differences in plant hormone, calcium ion, glucose metabolism, and photosynthesis pathway genes within the curved stem regions.	[203]

**Table 4:** Genes and their functions in the stem improvement of ornamental plants.

**Category**	**Gene**	**Species**	**Function**	**Reference**
Ethylene biosynthesis	*ACO1(0.821 kb)-ipt*	*Chrysanthemum* sp.	Transgenic lines exhibited increased branching and reduced internode lengths.	[209]
	*ACS1、 ACO1*	*Petunia hybrida*	*PhFBH4* positively regulates the transcription level of ethylene biosynthesis genes *ACS1* and *ACO1. PhFBH4-OX* plants showed shorter internodes and dwarf trait.	[210]
Gibberellin biosynthesis	*GA20ox*	*Brassica oleracea*	Jointly silencing *BoDWARF*, *BoGA20ox,* and *BoSP* produced a miniature plant.	[211]
	*GA2ox*	*Prunus Mume,* *Prunus armeniaca,* *Prunus salicina* *Prunus persica*	Conserved motif and gene structure analysis showed that *GAoxs* were conserved in the four Prunus species. Overexpression of *PmGA2ox8* in Arabidopsis leads to dwarfing phenotype.	[108]
	*GA20ox1* *GA2ox3*	*Jasminum sambac*	High GA_4_ level in elongating internode coincided with strong JsGA20ox1 and JsGAS1 expression in leaves, and JsGA2ox3 in internodes.	[104]
gibberellin signaling	*GAI*	*Petunia hybrida*	Overexpression of the gai mutant protein, which interferes with gibberellic acid signaling, leading to stunted growth and short internodes.	[212]
Lignin biosynthesis	*LAC4*	*Paeonia lactiflora*	*PlLAC4,* involved in lignin biosynthesis, positively regulates its deposition in herbaceous peony, enhancing stem mechanical strength.	[213]
	*HLB*	*Chrysanthemum morifolium*	*CmHLB* interacted with CmKNAT7, negatively regulates secondary cell wall formation, affecting stem mechanical strength in *Chrysanthemum*.	[214]
MAP kinase	*MKS1*	*Kalanchoe blossfeldiana*	Heterologous overexpression of *AtMKS1* result in a dwarf and delayed flowering phenotype.	[215]
Zinc-finger protein	LIF	*Petunia hybrida*	Transgenic petunia plant alters cytokinin metabolism and dramatic reduced plant height.	[216]
Carotenoid cleavage dioxygenase	CCD	*Petunia hybrida*	Loss of *PhCCD8* reduced internode length.	[217]
Transcription factor	SPL13A	*Lilium*	SPL13A regulates stem elongation in the adult vegetative phase	[218]
	WUSCEL-related homebox3 (WOX3)	*Panicum virgatum*	Overexpression of PvWOX3a increased stem length and internode diameter.	[133]
	WOX	*Melastoma dodecandrum*	*WOX* genes exhibited expression in the stem	[219]
	R2R3-MYBs	*Paeonia lactiflora*	R2R3-MYB TFs (PlMYB43, PlMYB83, PlMYB103) expressed in stem, regulated stem strength, cell wall thickness, and lignin deposition.	[213]
	WRKY41	*Paeonia lactiflora*	PlMYB43-PlWRKY41a complex activates PlXTH4 expression to enhance stem strength by adjusting secondary cell wall thickness.	[220]
	SRS7	*Chrysanthemum morifolium*	SRS7 overexpression downregulates GA-related genes and upregulates auxin genes, reducing internode length in transgenic pot-mums.	[221]
	SRS7	*Chrysanthemum* spp.	Overexpression of the BrSRS7 gene was previously shown to reduce the plant height of chrysanthemums	[222]
	SHI	*Kalanchoe blossfeldiana*	Transferring the Arabidopsis short internode (*shi*) gene into *Kalanchoe* resulted in a dwarf phenotype, reduced plant height and diameter.	[223]

Given the cellular complexity of developing stems, single-cell RNA-sequencing (scRNA-seq) and spatial transcriptomics technologies now provide unprecedented resolution to dissect distinct cell types and their spatial organization. By enabling high-resolution analysis of gene expression at the individual cell level, these approaches clarify cell-type-specific programs driving morphogenesis [204]. These techniques are particularly valuable for dissecting the cellular programs governing ornamental stem traits, such as internode elongation, vascular patterning, and secondary growth. For instance, scRNA-seq has been utilized to construct a developmental trajectory in the stem-differentiating xylem of *Liriodendron chinense* and *Trochodendron aralioides*, identifying specialized cell types like ray and fusiform initials and providing insights into xylem cell evolution [205].

Moreover, integrating the multilayered data, such as transcriptomic, metabolomic, and proteomic profiles, can reveal the interrelationships among gene expression, metabolite changes, and protein functions, constructing a systematic regulatory network for stem development (Table 3). Therefore, the application of omics technologies provides important clues for in-depth studies of stem development in ornamental plants.

### Genetic transformation advances in ornamental plant stem development

With the development of molecular technology innovations and biotechnology applications, such as transcriptome analysis, genome-wide association study (GWAS), and the clustered regularly interspaced short palindromic repeats (CRISPR)/CRISPR-associated protein 9 (Cas9) (CRISPR/Cas9) system, the functions of numerous genes and transcription factors in the regulation of stem development have been identified (Table 4). For example, a key *PmWEEP* gene was mapped in *Prunus mume* by GWAS [206]. The previous study showed that the silencing of *WEEP* resulted in more downward and wandering shoot orientations in *P. mume* [207]. Furthermore, the *WEEP* gene can promote negative gravitropism by establishing asymmetric auxin gradients [208].

In addition to strategies aimed at manipulating gene expression, the direct application of microbial systems also offers a route to inducing significant phenotypic changes in stem development. *Agrobacterium rhizogenes* carry a root-inducing (Ri) plasmid, which enables T-DNA integration carrying *rolA*, *rolB*, *rolC*, and *rolD* oncogenes into host plant genomes [224, 225]. Both *A. rhizogenes* and *rol*-transgenic plants presented hairy root phenotypes. Surprisingly, these transgenic plants always exhibit a dwarf phenotype, reduced apical dominance, and altered flowering habits. Horticultural applications exploit these phenotypic changes to create compact architecture in ornamental plants [226]. Compared to wild-type controls, *Pelargonium* sp. transformed with wild-type Ri plasmids presented 40% ~ 60% height reduction [227]. Similarly, the height of *Kalanchoe blossfeldiana* Ri-transgenic lines was reduced by 51.9% compared with that of control plants [228]. The overexpression of the rol gene in *K. blossfeldiana* also leads to dwarf and compact plants [229]. These morphological modifications increase ornamental value by improving plant architecture.

To achieve even greater precision and control on stem traits, recent advances have focused on directly editing key regulatory genes. CRISPR-Cas system, which originates from the bacterial immune system, serves as a gene-editing tool. A guide RNA (gRNA) directs the Cas9 enzyme to targeted DNA sites for cleavage, enabling precise gene editing [230]. This system has shown great potential in genetic breeding and trait improvement of horticultural crops, such as accelerating compact plant type breeding, optimizing stem quality, and improving lodging resistance, greatly shortening the breeding cycle of commercial flowers such as chrysanthemum, rose, and lily [231, 232] (Table 4).

## Future perspectives

### Advanced technologies for stem structure and cell wall characterization

Advances in computer vision and microscopic imaging technologies are enabling visualization and quantification of plant stem architecture and cell wall composition.

In stem phenotyping, micro-computed tomography (micro-CT) enables nondestructive, multilayered scanning, generating high-resolution three-dimensional (3D) visualization of stem internal anatomy, surpassing traditional paraffin sectioning and enabling tracking during whole growth process [233]. Coupled with vessel parser software, micro-CT allows recognize and quantitative analysis of different tissues, accurately parsing and calculating the shape, number, and distribution of vascular bundles in crops [234]. Advancing this approach, recent studies have integrated micro-CT with large-volume fully automated cell reconstruction (LVACR) were used to establish the multicellular morphological characteristics of plant organs, comprehensively displaying the fine structure and dynamic changes in plant tissues and organs [235]. This study provides profound insights into the precise spatial arrangement and cell behavior of multicellular organisms. Complementing these structural imaging techniques, virtual staining technology (VST) integrates (deep) machine learning, converting bright-field microscope images into virtual staining images highly consistent with actual fluorescence images, thereby achieving precise and label-free visualization of specific cells [236, 237]. Therefore, VST provides high-precision cell morphological data, offering a new tool for plant cell biology research.

Complementing structural imaging, frontier techniques are deepening insights into the nanoscale composition and mechanics of the plant cell wall. The main components of the cell wall include cellulose, hemicellulose, pectin, and lignin, which form a complex cross-linked network. This structure is a natural nanostructure critical for stem strength, elongation, and morphogenesis [238]. Although histochemical staining and fluorescence labeling have been the main tools for studying cell wall composition and structure, they lack the ability to observe dynamic changes in living cell walls in real time and are unable to accurately quantify multiple components. In contrast, emerging technologies enable dynamic, multimodal, and quantitative characterization of cell wall architecture.

Recently, nuclear magnetic resonance (NMR), atomic force microscopy (AFM), and spectral imaging techniques have further advanced the understanding of cell walls. NMR provides chemical information on cell wall components, AFM quantifies the mechanical properties of cell walls at the nanoscale, infrared spectral imaging maps the spatial distributions of components such as cellulose, hemicellulose, and lignin [239]. Confocal Raman microscopy (CRM) and stimulated Raman scattering (SRS) are two imaging techniques providing new tools for studying the microstructure of the cell wall. They can simultaneously obtain spectral and spatial data without damaging the sample and identify different components in the cell wall [240].

These technologies enable precise tracking of stem development and structural analysis, providing a robust foundation for breeding ornamental plants with improved stem quality, architecture, and mechanical performance.

### Precise design strategies for ornamental plants in the era of Breeding 5.0

With the continuous development of the global horticultural industry, ornamental plants are required to meet diverse and personalized market needs. Consumers now focus on shoot architecture, alongside traditional traits such as flower size and color. The ideal plant height is more convenient for transportation and commercial placement, while the stem thickness enhances lodging resistance in outdoor landscapes. Thus, the precise design and breeding of ornamental plant stem traits have become a key route for meeting the market demand in industrial upgrades.

The evolution of breeding methodologies has progressed through several generations, with Breeding 4.0 emphasizes the use of genomics, gene editing, and molecular marker–assisted breeding to achieve better varieties [241]. While Breeding 4.0 significantly improved breeding efficiency through molecular techniques, it still relies heavily on prior knowledge of gene functions and trait associations. This reliance limits its capacity to address complex, polygenic traits such as stem architecture in ornamental plants. Looking ahead, the design of ornamental plant stem traits will enter a new era, Breeding 5.0. Breeding 5.0 introduces the concept of intelligent breeding of smart varieties, integrating advanced technologies such as artificial intelligence (AI) and bioinformatics to achieve precise phenotypic design, including stem morphology. Breeding 5.0 aims to cultivate intelligent varieties with optimized resource utilization efficiency and high yield and quality [242].

**Figure 5:**
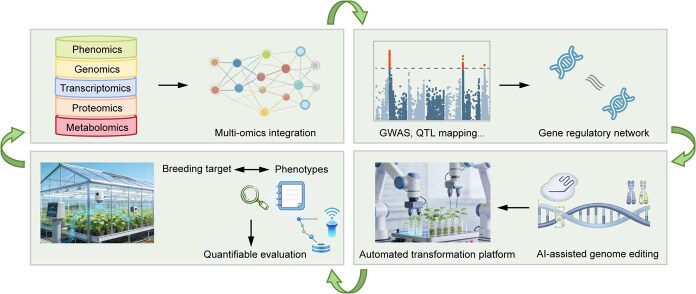
Strategies for smart ornamental plant breeding. A closed-loop smart breeding framework for ornamental plants that integrates high-resolution phenotyping with multi-omics profiling, leverages AI to decode genotype–phenotype relationships and prioritize editing targets, enables precision genome editing via automated platforms, and validates improved lines through dynamic monitoring in smart greenhouses to support iterative pipeline optimization.

To realize smart breeding in ornamental plants, we propose a framework integrating four key technological steps (Fig. 5). First, we establish a high-resolution phenotypic and omics database. Robotic platforms and multi-sensor imaging system capture real-time 3D plant architecture, physiological indexes, and micro-environmental data. At the same time, establish a multi-omics database, including pan-genomics, transcriptomics, proteomics, and metabolomics to profiles the molecular basis of key ornamental traits. Second, AI-aided predictive modeling is applied to construct developmental models and decode genotype-to-phenotype associations. Through AI-aided GWAS, key genetic loci and regulatory networks governing important ornamental characteristics are systematically identified. Third, guided by AI-designed CRISPR/Cas editing targets, precision breeding is implemented via automated genetic transformation platforms. This enables rapid development of improved lines tailored to specific breeding goals, such as enhancing vase life in cut flowers, compacting growth for vertical greening, and improving shade tolerance for indoor pot flowers. Finally, cultivation validation and dynamic phenotypic monitoring are performed in a smart greenhouse. These systems allow quantitative assessment of breeding objectives and phenotypic stability, generating closed-loop feedback data that drives the iterative optimization of the breeding pipeline (Fig. 5).

However, the commercialization of gene-edited ornamental plants still faces practical hurdles, including potential off-target effects of CRISPR/Cas systems, technical difficulties posed by genomic complexities such as high heterozygosity and polyploidy [243], and the challenge of coordinating multi-gene editing for complex polygenic traits like flower color, flower shape, and vase life [244], all of which pose significant quality control challenges for scalable production.

## References

[1] Zhang X, Su J, Jia F. et al. Genetic architecture and genomic prediction of plant height-related traits in chrysanthemum. Hortic Res. 2024b;11:uhad236 38222820 10.1093/hr/uhad236 PMC10782495

[2] Sanchez P, Nehlin L, Greb T. From thin to thick: major transitions during stem development. Trends Plant Sci. 2012;17:113–21 22189413 10.1016/j.tplants.2011.11.004 PMC3315019

[3] Yamaji N, Ma JF. Node-controlled allocation of mineral elements in *Poaceae*. Curr Opin Plant Biol. 2017;39:18–24 28558362 10.1016/j.pbi.2017.05.002

[4] Mekapogu M, Kwon O-K, Song H-Y. et al. Towards the improvement of ornamental attributes in *chrysanthemum*: recent progress in biotechnological advances. Int J Mol Sci. 2022;23:12284 36293140 10.3390/ijms232012284 PMC9603847

[5] Dinneny JR, Yanofsky MF. Vascular patterning: xylem or phloem? Curr Biol. 2004;14:R112–4 14986642

[6] Furuta KM, Hellmann E, Helariutta Y. Molecular control of cell specification and cell differentiation during procambial development. Annu Rev Plant Biol. 2014;65:607–38 24579995 10.1146/annurev-arplant-050213-040306

[7] Lewis AM . Plant stems: physiology and functional morphology. Tree Physiol. 1997;17:603–4

[8] Zanne AE, Falster DS. Plant functional traits-linkages among stem anatomy, plant performance and life history. New Phytol. 2010;185:348–51 20088975 10.1111/j.1469-8137.2009.03135.x

[9] Kulkarni AA, Thengane S, Krishnamurthy K. Direct shoot regeneration from node, internode, hypocotyl and embryo explants of *Withania somnifera*. Plant Cell Tissue Organ Cult. 2000;62:203–9

[10] Oka GM, Triwiyono A, Awaludin A. et al. Effects of node, internode and height position on the mechanical properties of *Gigantochloa atroviolacea* bamboo. Procedia Eng. 2014;95:31–7

[11] Falster DS, Westoby M. Plant height and evolutionary games. Trends Ecol Evol. 2003;18:337–43

[12] Fernandez MGS, Becraft PW, Yin Y. et al. From dwarves to giants? Plant height manipulation for biomass yield. Trends Plant Sci. 2009;14:454–61 19616467 10.1016/j.tplants.2009.06.005

[13] Shah DU, Reynolds TPS, Ramage MH. The strength of plants: theory and experimental methods to measure the mechanical properties of stems. J Exp Bot. 2017;68:4497–516 28981787 10.1093/jxb/erx245

[14] Galindo-Castaneda T, Hartmann M, Lynch JP. Location: root architecture structures rhizosphere microbial associations. J Exp Bot. 2024;75:594–604 37882632 10.1093/jxb/erad421 PMC10773995

[15] Iswanto ABB, Kang H, Park S. et al. Recent advances in improving yield and immunity through transcription factor engineering. J Integr Plant Biol. 2025;67:2005–27 40396540 10.1111/jipb.13932 PMC12315486

[16] Zhao D, de Voil, Sadras VO. et al. The plasticity of root traits and their effects on crop yield and yield stability. Plant Soil. 2025a;513:367–82

[17] He F, Liang T, Zhao R. et al. Modelling the lateral behavior of trunk-root-soil systems with representative branching patterns using a combined p-y framework. Comput Geotech. 2026;189:107654

[18] Bergstrand KJI . Methods for growth regulation of greenhouse produced ornamental pot- and bedding plants - a current review. Folia Hortic. 2017;29:63–74

[19] Neto AEF, Boldrin KVF, Mattson NS. Nutrition and quality in ornamental plants. Ornam Hortic. 2015;21:139–50

[20] Conway SJ, Drinnan AN. Analysis of surface growth in the conifer shoot apical meristem. Int J Plant Sci. 2017;178:273–87

[21] Serrano-Mislata A, Sablowski R. The pillars of land plants: new insights into stem development. Curr Opin Plant Biol. 2018;45:11–7 29763857 10.1016/j.pbi.2018.04.016 PMC6250904

[22] Bowman JL, Eshed Y. Formation and maintenance of the shoot apical meristem. Trends Plant Sci. 2000;5:110–5 10707076 10.1016/s1360-1385(00)01569-7

[23] Shapiro BE, Tobin C, Mjolsness E. et al. Analysis of cell division patterns in the *Arabidopsis* shoot apical meristem. Proc Natl Acad Sci USA. 2015;112:4815–20 25825722 10.1073/pnas.1502588112 PMC4403164

[24] Davière JM, Wild M, Regnault T. et al. Class I TCP-DELLA interactions in inflorescence shoot apex determine plant height. Curr Biol. 2014;24:1923–8 25127215 10.1016/j.cub.2014.07.012

[25] Leiboff S, Li X, Hu HC. et al. Genetic control of morphometric diversity in the maize shoot apical meristem. Nat Commun. 2015;6:8974 26584889 10.1038/ncomms9974 PMC4673881

[26] Moraes TS, Dornelas MC, Martinelli AP. FT/TFL1: calibrating plant architecture. Front Plant Sci. 2019;10:97 30815003 10.3389/fpls.2019.00097 PMC6381015

[27] Assefa T, Otyama PI, Brown AV. et al. Genome-wide associations and epistatic interactions for internode number, plant height, seed weight and seed yield in soybean. BMC Genomics. 2019;20:527 31242867 10.1186/s12864-019-5907-7 PMC6595607

[28] Hu JN, Li G, Mo HL. et al. Crop node detection and internode length estimation using an improved YOLOv5 model. Agriculture-Basel. 2023;13:473

[29] Wu C, Bai Y, Cao Z. et al. Plasticity in the morphology of growing bamboo: a bayesian analysis of exogenous treatment effects on plant height, internode length, and internode numbers. Plants. 2023;12:1713 37111934 10.3390/plants12081713 PMC10145155

[30] Gaur VS, Channappa G, Chakraborti M. et al. ‘Green revolution’ dwarf gene *sd1* of rice has gigantic impact. Brief Funct Genomics. 2020;19:390–409

[31] Nam KH, Li J. BRI1/BAK1, a receptor kinase pair mediating brassinosteroid signaling. Cell. 2002;110:203–12 12150928 10.1016/s0092-8674(02)00814-0

[32] Pan H, Zhou R, Louie GV. et al. Structural studies of cinnamoyl-CoA reductase and cinnamyl-alcohol dehydrogenase, key enzymes of monolignol biosynthesis. Plant Cell. 2014;26:3709–27 25217505 10.1105/tpc.114.127399 PMC4213152

[33] Ponniah SK, Shang ZH, Akbudak MA. et al. Down-regulation of hydroxycinnamoyl CoA: shikimate hydroxycinnamoyl transferase, cinnamoyl CoA reductase, and cinnamyl alcohol dehydrogenase leads to lignin reduction in rice (*Oryza sativa* L. *ssp. japonica cv. Nipponbare)*. Plant Biotechnol Rep. 2017;11:17–27

[34] Kumar M, Turner S. Plant cellulose synthesis: CESA proteins crossing kingdoms. Phytochemistry. 2015;112:91–9 25104231 10.1016/j.phytochem.2014.07.009

[35] Zhao Y, Wang X, Gao J. et al. The MYB61-STRONG2 module regulates culm diameter and lodging resistance in rice. J Integr Plant Biol. 2025b;67:243–57 39760479 10.1111/jipb.13830

[36] Herburger K, Frankova L, Picmanova M. et al. Hetero-transb-glucanase produces cellulose–xyloglucan covalent bonds in the cell walls of structural plant tissues and is stimulated by expansin. Mol Plant. 2020;13:1047–62 32376294 10.1016/j.molp.2020.04.011 PMC7339142

[37] Miedes E, Zarra I, Hoson T. et al. Xyloglucan endotransglucosylase and cell wall extensibility. J Plant Physiol. 2011;168:196–203 20828871 10.1016/j.jplph.2010.06.029

[38] Yuan W, Yao F, Liu Y. et al. Identification of the xyloglucan endotransglycosylase/hydrolase genes and the role of *PagXTH12* in drought resistance in poplar. For Res. 2024;4:0 10.48130/forres-0024-0036 PMC11870306 40027451

[39] Lu R, Zhang J, Wu YW. et al. bHLH transcription factors LP1 and LP2 regulate longitudinal cell elongation. Plant Physiol. 2021b;187:2577–91 34618066 10.1093/plphys/kiab387 PMC8644604

[40] Wu Q, Zhong S, Shi H. MicroProteins: dynamic and accurate regulation of protein activity. J Integr Plant Biol. 2022;64:812–20 35060666 10.1111/jipb.13229

[41] Zhong C, Patra B, Tang Y. et al. A transcriptional hub integrating gibberellin-brassinosteroid signals to promote seed germination in *Arabidopsis*. J Exp Bot. 2021;72:4708–20 33963401 10.1093/jxb/erab192 PMC8219041

[42] Awale P, McSteen P. Hormonal regulation of inflorescence and intercalary meristems in grasses. Curr Opin Plant Biol. 2023;76:102451 37739867 10.1016/j.pbi.2023.102451

[43] Yu KMJ, Oliver J, McKinley B. et al. *Bioenergy sorghum* stem growth regulation: intercalary meristem localization, development, and gene regulatory network analysis. Plant J. 2022;112:476–92 36038985 10.1111/tpj.15960

[44] Niu X, Fu D. The roles of BLH transcription factors in plant development and environmental response. Int J Mol Sci. 2022;23:3731 35409091 10.3390/ijms23073731 PMC8998993

[45] Tsuda K, Abraham-Juarez MJ, Maeno A. et al. KNOTTED1 cofactors, BLH12 and BLH14, regulate internode patterning and vein anastomosis in maize. Plant Cell. 2017;29:1105–18 28381444 10.1105/tpc.16.00967 PMC5466031

[46] Baucher M, El Jaziri M, Vandeputte O. From primary to secondary growth: origin and development of the vascular system. J Exp Bot. 2007;58:3485–501 17898423 10.1093/jxb/erm185

[47] Du J, Wang YC, Chen WF. et al. High-resolution anatomical and spatial transcriptome analyses reveal two types of meristematic cell pools within the secondary vascular tissue of poplar stem. Mol Plant. 2023;16:809–28 36895162 10.1016/j.molp.2023.03.005

[48] Tang Z, Liang YQ, Wang ML. et al. Effect of mechanical properties of rice stem and its fiber on the strength of straw rope. Ind Crop Prod. 2022;180:114729

[49] Cosgrove DJ, Li LC, Cho HT. et al. The growing world of expansins. Plant Cell Physiol. 2002;43:1436–44 12514240 10.1093/pcp/pcf180

[50] Somerville C . Cellulose synthesis in higher plants. Annu Rev Cell Dev Biol. 2006;22:53–78 16824006 10.1146/annurev.cellbio.22.022206.160206

[51] Saxena IM, Brown RM Jr. Cellulose biosynthesis: current views and evolving concepts. Ann Bot. 2005;96:9–21 15894551 10.1093/aob/mci155 PMC4246814

[52] Taylor NG, Howells RM, Huttly AK. et al. Interactions among three distinct CesA proteins essential for cellulose synthesis. Proc Natl Acad Sci USA. 2003;100:1450–5 12538856 10.1073/pnas.0337628100 PMC298793

[53] Huang Y, Ren A, Wan Y. et al. Effect of the pectin contents and nanostructure on the stem straightness of two *Paeonia lactiflora* cultivars. PeerJ. 2023;11:e15166 37073273 10.7717/peerj.15166 PMC10106084

[54] Zhao H, Wang S, Yang R. et al. Side chain of confined xylan affects cellulose integrity leading to bending stem with reduced mechanical strength in ornamental plants. Carbohydr Polym. 2024;329:121787 38286554 10.1016/j.carbpol.2024.121787

[55] Hayashi T, Kaida R. Functions of xyloglucan in plant cells. Mol Plant. 2011;4:17–24 20943810 10.1093/mp/ssq063

[56] Hu H, Zhang R, Zhao Y. et al. Cell wall remodeling confers plant architecture with distinct wall structure in *Nelumbo nucifera*. Plant J. 2024a;120:1392–409 39427333 10.1111/tpj.17056

[57] Yang Y, Huang Y, Ren A. et al. Xylem development and phloem conductivity in relation to the stem mechanical strength of *Paeonia lactiflora*. J Plant Physiol. 2023;283:153963 36905700 10.1016/j.jplph.2023.153963

[58] Zhao D, Hao Z, Tao J. Effects of shade on plant growth and flower quality in the herbaceous peony (*Paeonia lactiflora* pall.). Plant Physiol Biochem. 2012;61:187–96 23141672 10.1016/j.plaphy.2012.10.005

[59] Ye Z . Association of caffeoyl coenzyme a 3-O-methyltransferase expression with lignifying tissues in several dicot plants. Plant Physiol. 1997;115:1341–50 9414548 10.1104/pp.115.4.1341 PMC158599

[60] Wolf S, Hematy K, Hofte H. Growth control and cell wall signaling in plants. Annu Rev Plant Biol. 2012;63:381–407 22224451 10.1146/annurev-arplant-042811-105449

[61] Philosoph-Hadas S, Meir S, Rosenberger I. et al. Regulation of the gravitropic response and ethylene biosynthesis in gravistimulated snapdragon spikes by calcium chelators and ethylene inhibitors. Plant Physiol. 1996;110:301–10 11536726 10.1104/pp.110.1.301 PMC157721

[62] García-González A, Soriano-Melgar L d AA, Cid-López ML. et al. Effects of calcium oxide nanoparticles on vase life of gerbera cut flowers. Sci Hortic. 2022;291:110532

[63] Li C, Tao J, Zhao D. et al. Effect of calcium sprays on mechanical strength and cell wall fractions of herbaceous peony (*Paeonia lactiflora* pall.) inflorescence stems. Int J Mol Sci. 2012;13:4704–13 22606005 10.3390/ijms13044704 PMC3344241

[64] Yu X, Lu G, Cheng F. et al. Effect of calcium on the stem quality of cut herbaceous peony. J Hunan AgricUniv (Nat Sci). 2011;36:531–5

[65] Sheng H, Chen S. Plant silicon-cell wall complexes: identification, model of covalent bond formation and biofunction. Plant Physiol Biochem. 2020;155:13–9 32736240 10.1016/j.plaphy.2020.07.020

[66] Luyckx M, Hausman JF, Lutts S. et al. Impact of silicon in plant biomass production: focus on bast fibres, hypotheses, and perspectives. Plants (Basel). 2017;6:37 28891950 10.3390/plants6030037 PMC5620593

[67] Zhao D, Xu C, Luan Y. et al. Silicon enhances stem strength by promoting lignin accumulation in herbaceous peony (*Paeonia lactiflora* pall.). Int J Biol Macromol. 2021;190:769–79 34520779 10.1016/j.ijbiomac.2021.09.016

[68] Jin Z, Li P, Huang R. et al. Natural variation in PtobZIP18 confers the trade-off between stem growth and drought tolerance in Populus. Plant Biotechnol J. 2025;23:4633–49 40652547 10.1111/pbi.70261 PMC12483958

[69] Shi L, Tu F, Yang J. Mechanical behaviors of porous bionic structure of lotus stem. Int J Solids Struct. 2024;290:112665

[70] Zou M, Xu S, Wei C. et al. A bionic method for the crashworthiness design of thin-walled structures inspired by bamboo. Thin-Walled Struct. 2016;101:222–30

[71] Lou T, Lv S, Wang J. et al. Cell size and xylem differentiation regulating genes from Salicornia europaea contribute to plant salt tolerance. Plant Cell Environ. 2024;47:2640–59 38558078 10.1111/pce.14905

[72] Wei Q, He M, Hu X. Effects of exogenous brassinolide on growth and physiological characteristics of *Matthiola incana* L.seedlings under salt stress. Southwest China J Agric Sci. 2023;36:1165–71

[73] Li X, Tian X, Li W. Effects of EBR and MT on the photosynthetic physiology of lily seedlings under salt alkali stress. Northern Hortic. 2024a;19:52–61

[74] Litwińczuk W, Jacek B. Growth of *Paulownia* ssp. interspecific hybrid ‘Oxytree’ micropropagated nursery plants under the influence of plant-growth regulators. Agronomy. 2023;13:2474

[75] Zhao W, Dong H, Hou H. et al. Establishment of a highly efficient In vitro regeneration system for *Rhododendron aureum*. Forests. 2023;14:1335

[76] Çelikel FG, Zhang Q, Zhang Y. et al. A cytokinin analog thidiazuron suppresses shoot growth in potted rose plants via the gibberellic acid pathway. Front Plant Sci. 2021;12:639717 34335639 10.3389/fpls.2021.639717 PMC8320663

[77] Yari V, Roein Z, Sabouri A. Exogenous 5-azaCitidine accelerates flowering and external GA (3) increases ornamental value in Iranian *anemone* accessions. Sci Rep. 2021;11:7478 33820923 10.1038/s41598-021-86940-6 PMC8021551

[78] Cornea-Cipcigan M, Pamfil D, Sisea CR. et al. Gibberellic acid can improve seed germination and ornamental quality of selected *cyclamen* species grown under short and long days. Agronomy. 2020;10:516

[79] Lee HB, Im NH, An SK. et al. Changes of growth and inflorescence initiation by exogenous gibberellic Acid3 and 6-benzylaminopurine application in *Phalaenopsis* orchids. Agronomy. 2021a;11:196

[80] Ranwala AP, Legnani G, Miller WB. Minimizing stem elongation during spray applications of gibberellin_4+7_ and benzyladenine to prevent leaf chlorosis in easter lilies. HortScience. 2003;38:1210–3

[81] Oh W, Kim KS. Light intensity and temperature regulate petiole elongation by controlling the content of and sensitivity to gibberellin in *Cyclamen persicum*. Hortic Environ Biotechnol. 2014;55:175–82

[82] Al-Khassawneh NM, Karam NS, Shibli RA. Growth and flowering of black iris (Dinsm.) following treatment with plant growth regulators. Sci Hortic. 2006;107:187–93

[83] Zhang D, Ren L, Yue JH. et al. RNA-Seq-based transcriptome analysis of stem development and dwarfing regulation in *Agapanthus praecox* ssp. orientalis (Leighton) Leighton. Gene. 2015;565:252–67 25865295 10.1016/j.gene.2015.04.013

[84] Zhu X, Chai M, Li Y. et al. Global transcriptome profiling analysis of inhibitory effects of paclobutrazol on leaf growth in lily (*Lilium Longiflorum*-*Asiatic* hybrid). Front Plant Sci. 2016;7:491 27148316 10.3389/fpls.2016.00491 PMC4835717

[85] Bálint J, Benedek K, Csorba AB. Assessing the effect of plant growth stimulants and retardants on *Cyclamen* “Halios F1 Salmon rose” cultivar. Horticulturae. 2024;10:53

[86] Ding H, Liu J, Wang W. et al. Dwarfing effect of uniconazole on *Curcuma alismatifolia* and its tissue anatomy. Acta Agric Zhejiangensis. 2013;25:975–9

[87] Zhao D, Luan Y, Shi W. et al. Melatonin enhances stem strength by increasing lignin content and secondary cell wall thickness in herbaceous peony. J Exp Bot. 2022a;73:5974–91 35436332 10.1093/jxb/erac165

[88] Sahraei F, Solgi M, Taghizadeh M. The application of methyl jasmonate in combination with ascorbic acid on morphological traits and some biochemical parameters in red willow. Physiol Mol Biol Plants. 2023;29:185–93 36875731 10.1007/s12298-023-01284-x PMC9981849

[89] Currey CJ, Lopez RG, Krug BA. et al. Substrate drenches containing flurprimidol suppress height of ‘Nellie White’ easter lilies. HortTech. 2012;22:164–8

[90] Fischer U, Kucukoglu M, Helariutta Y. et al. The dynamics of cambial stem cell activity. Annu Rev Plant Biol. 2019;70:293–319 30822110 10.1146/annurev-arplant-050718-100402

[91] Wang D, Chen Y, Li W. et al. Vascular cambium: the source of wood formation. Front Plant Sci. 2021;12:700928 34484265 10.3389/fpls.2021.700928 PMC8416278

[92] Ji Y, Xue L, Lei J. Regulation of scape elongation through gibberellin and auxin detected by hormone metabolomic profiling in *Clivia miniata*. Ornam Plant Res. 2025;5:e041

[93] Ljung K, Bhalerao RP, Sandberg G. Sites and homeostatic control of auxin biosynthesis in *Arabidopsis* during vegetative growth. Plant J. 2001;28:465–74 11737783 10.1046/j.1365-313x.2001.01173.x

[94] Makila R, Wybouw B, Smetana O. et al. Gibberellins promote polar auxin transport to regulate stem cell fate decisions in cambium. Nat Plants. 2023;9:631–44 36997686 10.1038/s41477-023-01360-w PMC10119023

[95] Smetana O, Makila R, Lyu M. et al. High levels of auxin signalling define the stem-cell organizer of the vascular cambium. Nature. 2019;565:485–9 30626967 10.1038/s41586-018-0837-0

[96] Vanneste S, Pei Y, Friml J. Mechanisms of auxin action in plant growth and development. Nat Rev Mol Cell Biol. 2025;26:648–66 40389696 10.1038/s41580-025-00851-2

[97] Wei T, Zhang L, Zhu R. et al. A gain-of-function mutant of IAA7 inhibits stem elongation by transcriptional repression of EXPA5 genes in Brassica napus. Int J Mol Sci. 2021;22:9018 34445724 10.3390/ijms22169018 PMC8396470

[98] Zheng M, Hu M, Yang H. et al. Three BnaIAA7 homologs are involved in auxin/brassinosteroid-mediated plant morphogenesis in rapeseed (Brassica napus L.). Plant Cell Rep. 2019;38:883–97 31011789 10.1007/s00299-019-02410-4 PMC6647246

[99] Daviere JM, Achard P. Gibberellin signaling in plants. Development. 2013;140:1147–51 23444347 10.1242/dev.087650

[100] Yamaguchi S . Gibberellin metabolism and its regulation. Annu Rev Plant Biol. 2008;59:225–51 18173378 10.1146/annurev.arplant.59.032607.092804

[101] Hedden P . The current status of research on gibberellin biosynthesis. Plant Cell Physiol. 2020;61:1832–49 32652020 10.1093/pcp/pcaa092 PMC7758035

[102] Lange T, Pimenta Lange MJ. The multifunctional dioxygenases of gibberellin synthesis. Plant Cell Physiol. 2020;61:1869–79 32343806 10.1093/pcp/pcaa051

[103] Xu H, Liu Q, Yao T. et al. Shedding light on integrative GA signaling. Curr Opin Plant Biol. 2014;21:89–95 25061896 10.1016/j.pbi.2014.06.010

[104] Zhang H, Wang W, Huang J. et al. Role of gibberellin and its three GID1 receptors in *Jasminum sambac* stem elongation and flowering. Planta. 2021b;255:17 34889996 10.1007/s00425-021-03805-y

[105] Xie Q, Chen G, Liu Q. et al. Dual silencing of *DmCPD* and *DmGA20ox* genes generates a novel miniature and delayed-flowering *Dendranthema morifolium* variety. Mol Breed. 2015;35:67

[106] Zhang X, Ding L, Song A. et al. DWARF AND ROBUST PLANT regulates plant height via modulating gibberellin biosynthesis in chrysanthemum. Plant Physiol. 2022;190:2484–500 36214637 10.1093/plphys/kiac437 PMC9706434

[107] Tong Z, Hong B, Yang Y. et al. Overexpression of two chrysanthemum DgDREB1 group genes causing delayed flowering or dwarfism in *Arabidopsis*. Plant Mol Biol. 2009;71:115–29 19544047 10.1007/s11103-009-9513-y

[108] Li X, Zhang J, Guo X. et al. Genome-wide analysis of the gibberellin-oxidases family members in four *Prunus* species and a functional analysis of *PmGA2ox8* in plant height. Int J Mol Sci. 2024b;25:8697 39201381 10.3390/ijms25168697 PMC11354515

[109] Willige BC, Ghosh S, Nill C. et al. The DELLA domain of GA INSENSITIVE mediates the interaction with the GA INSENSITIVE DWARF1A gibberellin receptor of *Arabidopsis*. Plant Cell. 2007;19:1209–20 17416730 10.1105/tpc.107.051441 PMC1913748

[110] Griffiths J, Murase K, Rieu I. et al. Genetic characterization and functional analysis of the GID1 gibberellin receptors in *Arabidopsis*. Plant Cell. 2006;18:3399–414 17194763 10.1105/tpc.106.047415 PMC1785415

[111] Wen C, Chang C. *Arabidopsis* RGL1 encodes a negative regulator of gibberellin responses. Plant Cell. 2002;14:87–100 11826301 10.1105/tpc.010325 PMC150553

[112] Petty LM, Harberd NP, Carré IA. et al. Expression of the *Arabidopsis gai* gene under its own promoter causes a reduction in plant height in chrysanthemum by attenuation of the gibberellin response. Plant Sci. 2003;164:175–82

[113] Chaudhary A, Hsiao YC, Jessica Yeh FL. et al. FERONIA signaling maintains cell wall integrity during brassinosteroid-induced cell expansion in *Arabidopsis*. Mol Plant. 2025;18:603–18 39916326 10.1016/j.molp.2025.02.001 PMC11981838

[114] Wang B, Smith SM, Li J. Genetic regulation of shoot architecture. Annu Rev Plant Biol. 2018;69:437–68 29553800 10.1146/annurev-arplant-042817-040422

[115] Guo H, Li L, Ye H. et al. Three related receptor-like kinases are required for optimal cell elongation in *Arabidopsis thaliana*. Proc Natl Acad Sci USA. 2009;106:7648–53 19383785 10.1073/pnas.0812346106 PMC2678668

[116] Choe S, Dilkes BP, Gregory BD. et al. The *Arabidopsis* dwarf1 mutant is defective in the conversion of 24-methylenecholesterol to campesterol in brassinosteroid biosynthesis. Plant Physiol. 1999;119:897–908 10069828 10.1104/pp.119.3.897 PMC32104

[117] Hong Z, Ueguchi-Tanaka M, Fujioka S. et al. The Rice brassinosteroid-deficient dwarf2 mutant, defective in the rice homolog of Arabidopsis DIMINUTO/DWARF1, is rescued by the endogenously accumulated alternative bioactive brassinosteroid, dolichosterone. Plant Cell. 2005;17:2243–54 15994910 10.1105/tpc.105.030973 PMC1182486

[118] Yin Y, Wang ZY, Mora-Garcia S. et al. BES1 accumulates in the nucleus in response to brassinosteroids to regulate gene expression and promote stem elongation. Cell. 2002;109:181–91 12007405 10.1016/s0092-8674(02)00721-3

[119] Brackmann K, Qi J, Gebert M. et al. Spatial specificity of auxin responses coordinates wood formation. Nat Commun. 2018;9:875 29491423 10.1038/s41467-018-03256-2 PMC5830446

[120] Bai MY, Shang JX, Oh E. et al. Brassinosteroid, gibberellin and phytochrome impinge on a common transcription module in Arabidopsis. Nat Cell Biol. 2012;14:810–7 22820377 10.1038/ncb2546 PMC3606816

[121] Liu K, Li Y, Chen X. et al. ERF72 interacts with ARF6 and BZR1 to regulate hypocotyl elongation in *Arabidopsis*. J Exp Bot. 2018b;69:3933–47 29897568 10.1093/jxb/ery220 PMC6054149

[122] Lee J, Kim H, Park SG. et al. Brassinosteroid-BZR1/2-WAT1 module determines the high level of auxin signalling in vascular cambium during wood formation. New Phytol. 2021b;230:1503–16 33570747 10.1111/nph.17265

[123] Hou L, Zhu L, Hao M. et al. Brassinosteroids enhance gibberellic acid biosynthesis to promote cotton fibre cell elongation. Plant Biotechnol J. 2025;23:1213–5 39853663 10.1111/pbi.14579 PMC11933871

[124] Tong H, Xiao Y, Liu D. et al. Brassinosteroid regulates cell elongation by modulating gibberellin metabolism in rice. Plant Cell. 2014;26:4376–93 25371548 10.1105/tpc.114.132092 PMC4277228

[125] Unterholzner SJ, Rozhon W, Papacek M. et al. Brassinosteroids are master regulators of gibberellin biosynthesis in *Arabidopsis*. Plant Cell. 2015;27:2261–72 26243314 10.1105/tpc.15.00433 PMC4568508

[126] Hu J, Su H, Cao H. et al. AUXIN RESPONSE FACTOR7 integrates gibberellin and auxin signaling via interactions between DELLA and AUX/IAA proteins to regulate cambial activity in poplar. Plant Cell. 2022;34:2688–707 35435234 10.1093/plcell/koac107 PMC9252472

[127] Serivichyaswat PT, Kareem A, Feng M. et al. Auxin signaling in the cambium promotes tissue adhesion and vascular formation during Arabidopsis graft healing. Plant Physiol. 2024;196:754–62 38701036 10.1093/plphys/kiae257 PMC11444275

[128] Zhang Y, Wang L, Wu Y. et al. Gibberellin promotes cambium reestablishment during secondary vascular tissue regeneration after girdling in an auxin-dependent manner in *Populus*. J Integr Plant Biol. 2024c;66:86–102 38051026 10.1111/jipb.13591

[129] Yu Q, Cheng C, Zhou X. et al. Ethylene controls cambium stem cell activity via promoting local auxin biosynthesis. New Phytol. 2023b;239:964–78 37282811 10.1111/nph.19004

[130] Cammarata J, Morales Farfan C, Scanlon MJ. et al. Cytokinin-CLAVATA cross-talk is an ancient mechanism regulating shoot meristem homeostasis in land plants. Proc Natl Acad Sci USA. 2022;119:e2116860119 35344421 10.1073/pnas.2116860119 PMC9168927

[131] Wang J, Tian C, Zhang C. et al. Cytokinin signaling activates WUSCHEL expression during axillary meristem initiation. Plant Cell. 2017;29:1373–87 28576845 10.1105/tpc.16.00579 PMC5502442

[132] Liu H, Zhang H, Dong YX. et al. DNA METHYLTRANSFERASE1-mediated shoot regeneration is regulated by cytokinin-induced cell cycle in *Arabidopsis*. New Phytol. 2018a;217:219–32 28960381 10.1111/nph.14814

[133] Yang R, Wu Z, Bai C. et al. Overexpression of PvWOX3a in switchgrass promotes stem development and increases plant height. Hortic Res. 2021;8:252 34848686 10.1038/s41438-021-00678-w PMC8633294

[134] Jia KP, Luo Q, He SB. et al. Strigolactone-regulated hypocotyl elongation is dependent on cryptochrome and phytochrome signaling pathways in *Arabidopsis*. Mol Plant. 2014;7:528–40 24126495 10.1093/mp/sst093

[135] Agusti J, Herold S, Schwarz M. et al. Strigolactone signaling is required for auxin-dependent stimulation of secondary growth in plants. Proc Natl Acad Sci USA. 2011;108:20242–7 22123958 10.1073/pnas.1111902108 PMC3250165

[136] Sang D, Chen D, Liu G. et al. Strigolactones regulate rice tiller angle by attenuating shoot gravitropism through inhibiting auxin biosynthesis. Proc Natl Acad Sci USA. 2014;111:11199–204 25028496 10.1073/pnas.1411859111 PMC4121785

[137] Ma Q, Wang X, Sun J. et al. Coordinated regulation of hypocotyl cell elongation by light and ethylene through a microtubule destabilizing protein. Plant Physiol. 2018;176:678–90 29167353 10.1104/pp.17.01109 PMC5761786

[138] Yang C, Lu X, Ma B. et al. Ethylene signaling in rice and *Arabidopsis*: conserved and diverged aspects. Mol Plant. 2015;8:495–505 25732590 10.1016/j.molp.2015.01.003

[139] Hattori Y, Nagai K, Furukawa S. et al. The ethylene response factors SNORKEL1 and SNORKEL2 allow rice to adapt to deep water. Nature. 2009;460:1026–30 19693083 10.1038/nature08258

[140] Kuroha T, Nagai K, Gamuyao R. et al. Ethylene-gibberellin signaling underlies adaptation of rice to periodic flooding. Science. 2018;361:181–6 30002253 10.1126/science.aat1577

[141] Lyu Y, Dong X, Niu S. et al. An orchestrated ethylene-gibberellin signaling cascade contributes to mesocotyl elongation and emergence of rice direct seeding. J Integr Plant Biol. 2024;66:1427–39 38751025 10.1111/jipb.13671

[142] Hatfield JL, Prueger JH. Temperature extremes: effect on plant growth and development. Weather Clim Extremes. 2015;10:4–10

[143] Luo X, Liu X, Zheng N. et al. Molecular mechanisms of temperature-mediated flowering regulation: from Arabidopsis to short-day crops. Plant Cell Environ. 2025;48:7020–37 40503598 10.1111/pce.15678

[144] Lu HP, Wang JJ, Wang MJ. et al. Roles of plant hormones in thermomorphogenesis. Stress Biol. 2021a;1:20 37676335 10.1007/s44154-021-00022-1 PMC10441977

[145] Bayer A, Ruter J, van Iersel MW. Elongation of *Hibiscus acetosella* under well-watered and drought-stressed conditions. HortScience. 2016;51:1384–8

[146] Alvarez S, Sanchez-Blanco MJ. Comparison of individual and combined effects of salinity and deficit irrigation on physiological, nutritional and ornamental aspects of tolerance in *Callistemon laevis* plants. J Plant Physiol. 2015;185:65–74 26277754 10.1016/j.jplph.2015.07.009

[147] Liu T, Cheng P, Zhao K. et al. Effects of blue light treatment on growth of summer-flowering *chrysanthemum* “Yuuka” tissue culture seedlings and its molecular mechanism. Jiangsu Agric Sci. 2022;50:150–6

[148] Roh YS, Yoo YK. Light quality of light emitting diodes affects growth, chlorophyll fluorescence and phytohormones of Tulip ‘Lasergame’. Hortic Environ Biotechnol. 2023;64:245–55

[149] Nissim-Levi A, Kitron M, Nishri Y. et al. Effects of blue and red LED lights on growth and flowering of *Chrysanthemum morifolium*. Sci Hortic. 2019;254:77–83

[150] Takemura Y, Kuroki K, Katou M. et al. Gene expression changes triggered by end-of-day far-red light treatment on early developmental stages of *Eustoma grandiflorum* (Raf.) Shinn. Sci Rep. 2015;5:17864 26642764 10.1038/srep17864 PMC4672308

[151] Islam MA, Tarkowská D, Clarke JL. et al. Impact of end-of-day red and far-red light on plant morphology and hormone physiology of poinsettia. Sci Hortic. 2014;174:77–86

[152] Gautam P, Terfa MT, Olsen JE. et al. Red and blue light effects on morphology and flowering of *petunia*×*hybrida*. Sci Hortic. 2015;184:171–8

[153] Zhang M, Park Y, Runkle ES. Regulation of extension growth and flowering of seedlings by blue radiation and the red to far-red ratio of sole-source lighting. Sci Hortic. 2020;272:109478

[154] Han S, Zhu XQ, Pei DL. Research of *Chrysanthemum morifolium* in morphology, tissue structure and gene expression. J Sichuan Agric Univ. 2019;37:34–40

[155] Su D, Fang Z, Li YL. et al. Effect of different NH4+-N/NO3-—N ratios on the growth of *Lilium*. Northern Hortic. 2012;02:67–9.

[156] Mortensen LM . Nitrogen oxides produced during CO_2_ enrichment. New Phytol. 1985;101:103–8 33873831 10.1111/j.1469-8137.1985.tb02819.x

[157] Dikbas N, Parlakova Karagoz F, Ucar S. et al. Ornamental cabbage (*Brassica oleracea* var. *acephala*) responses to phytase enzyme purified from *lactobacillus coryniformis* application. Biotechnol Appl Biochem. 2023;70:1407–20 36779503 10.1002/bab.2449

[158] Kamenidou S, Cavins TJ, Marek SM. Silicon supplements affect horticultural traits of greenhouse-produced ornamental sunflowers. HortScience. 2008;43:236–9

[159] Fan HM, Wang XW, Sun X. et al. Effects of humic acid derived from sediments on growth, photosynthesis and chloroplast ultrastructure in chrysanthemum. Sci Hortic. 2014;177:118–23

[160] Mata DA, Botto JF. Photoperiod, light, and temperature requirements to control plant architecture and flowering time in *Salvia exserta*. J Hortic Sci Biotechnol. 2011;86:408–14

[161] Wu SZ, Lu JL, Li H. et al. The effects of variable temperature treatment on the growth and development of lily (*Lilium* spp.) and the control of *Rhizoglyphus echinopus*. J Yangzhou Univ. 2021;42:87–91

[162] Chen C . Evaluation of the effect of temperature on a stem elongation model of *Phalaenopsis*. Horticulturae. 2019;5:76

[163] Schouten RE, Carvalho SM, Heuvelink E. et al. Modelling of temperature-controlled internode elongation applied to chrysanthemum. Ann Bot. 2002;90:353–9 12234147 10.1093/aob/mcf196 PMC4240396

[164] Carvalho SM, Heuvelink E, Cascais R. et al. Effect of day and night temperature on internode and stem length in chrysanthemum: is everything explained by DIF? Ann Bot. 2002;90:111–8 12125764 10.1093/aob/mcf154 PMC4233858

[165] Van Der Ploeg A, Heuvelink E. The influence of temperature on growth and development of chrysanthemum cultivars. J Hortic Sci Biotechnol. 2006;81:174–82

[166] Jing Y, Lin R. Transcriptional regulatory network of the light signaling pathways. New Phytol. 2020;227:683–97 32289880 10.1111/nph.16602

[167] Kulchin YN, Nakonechnaya OV, Gafitskaya IV. et al. Plant morphogenesis under different light intensity. Defect Diffus Forum. 2018;386:201–6

[168] Osnato M, Cota I, Nebhnani P. et al. Photoperiod control of plant growth: flowering time genes beyond flowering. Front Plant Sci. 2021;12:805635 35222453 10.3389/fpls.2021.805635 PMC8864088

[169] Yang XL, Xu H, Shao L. et al. Response of photosynthetic capacity of tomato leaves to different LED light wavelength. Environ Exp Bot. 2018;150:161–71

[170] Vermeulen PJ, Anten NPR, Schieving F. et al. Height convergence in response to neighbour growth: genotypic differences in the stoloniferous plant *Potentilla reptans*. New Phytol. 2008;177:688–97 18069962 10.1111/j.1469-8137.2007.02301.x

[171] Liu Y, Jafari F, Wang H. Integration of light and hormone signaling pathways in the regulation of plant shade avoidance syndrome. aBIOTECH. 2021;2:131–45 36304753 10.1007/s42994-021-00038-1 PMC9590540

[172] Martel AB, Taylor AE, Qaderi MM. Individual and interactive effects of temperature and light intensity on canola growth, physiological characteristics and methane emissions. Plant Physiol Biochem. 2020;157:160–8 33120108 10.1016/j.plaphy.2020.10.016

[173] Rondanini DP, del Pilar Vilariño, Roberts ME. et al. Physiological responses of spring rapeseed (*Brassica napus*) to red/far-red ratios and irradiance during pre- and post-flowering stages. Physiol Plant. 2014;152:784–94 24814241 10.1111/ppl.12227

[174] Su P, Wang D, Wang P. et al. In vitro regeneration, photomorphogenesis and light signaling gene expression in *Hydrangea quercifolia* cv. 'Harmony' under different LED environments. Planta. 2024;259:71 38353793 10.1007/s00425-024-04335-z

[175] Sharma P, Chatterjee M, Burman N. et al. Cryptochrome 1 regulates growth and development in *brassica* through alteration in the expression of genes involved in light, phytohormone and stress signalling. Plant Cell Environ. 2014;37:961–77 24117455 10.1111/pce.12212

[176] Folta KM . Green light stimulates early stem elongation, antagonizing light-mediated growth inhibition. Plant Physiol. 2004;135:1407–16 15247396 10.1104/pp.104.038893 PMC519058

[177] Zheng Z-L, Yang Z, Jang J-C. et al. Modification of plant architecture in chrysanthemum by ectopic expression of the tobacco phytochrome B1 gene. J Am Soc Hortic Sci. 2001;126:19–26

[178] Jeong SW, Park S, Jin JS. et al. Influences of four different light-emitting diode lights on flowering and polyphenol variations in the leaves of chrysanthemum (*Chrysanthemum morifolium*). J Agric Food Chem. 2012;60:9793–800 22970652 10.1021/jf302272x

[179] Molla KA . A significant P value: how phosphorus controls plant height. Plant Cell. 2024;36:213–4 37943675 10.1093/plcell/koad284 PMC10827309

[180] Alem P, Thomas PA, van Iersel MW. Controlled water deficit as an alternative to plant growth retardants for regulation of poinsettia stem elongation. HortScience. 2015;50:565–9

[181] Bayer A . Fertilizer rate and substrate water content effect on growth and flowering of *beardtongue*. Horticulturae. 2020;6:57

[182] Pimentel P, Almada RD, Salvatierra A. et al. Physiological and morphological responses of *Prunus* species with different degree of tolerance to long-term root hypoxia. Sci Hortic. 2014;180:14–23

[183] Nzokou P, Cregg B. Growth, Biomass, and Nitrogen Use Efficiency of Containerized Fraser Fir (Abies fraseri) as Related to Irrigation and Nitrogen Fertilization. *HortScience horts*. 2010, 45: 946-951

[184] Li W, Wang Y., Xie Z Xie Z. *et al*. Effects of Different Irrigation Amount on Growth and Yield of Lanzhou Lily. *North Hortic*. 2020;11:54-63.

[185] Caspersen S, Bergstrand K-J. Phosphorus restriction influences P efficiency and ornamental quality of poinsettia and chrysanthemum. Sci Hortic. 2020;267:109316

[186] Boldt JK, Altland JE. Petunia (petunia ×hybrida) cultivars vary in silicon accumulation and distribution. HortScience. 2021;56:305-+

[187] Jiang KF, Peng S, Yin ZM. et al. Effects of N, P, K nutrition levels on the growth, flowering attributes and functional components in *Chrysanthemum morifolium*. Horticulturae. 2024;10:226

[188] Kamenidou S, Cavins TJ, Marek S. Silicon supplements affect floricultural quality traits and elemental nutrient concentrations of greenhouse produced gerbera. Sci Hortic. 2010;123:390–4

[189] Lian X, Tan B, Yan L. et al. Transcript profiling provides insights into molecular processes during shoot elongation in temperature-sensitive peach (*Prunus persica*). Sci Rep. 2020;10:7801 32385278 10.1038/s41598-020-63952-2 PMC7210264

[190] Testone G, Condello E, Verde I. et al. The peach (*Prunus persica* L. Batsch) genome harbours 10 KNOX genes, which are differentially expressed in stem development, and the class 1 KNOPE1 regulates elongation and lignification during primary growth. J Exp Bot. 2012;63:5417–35 22888130 10.1093/jxb/ers194 PMC3444263

[191] Zhang Q, Chen W, Sun L. et al. The genome of *Prunus mume*. Nat Commun. 2012;3:1318 23271652 10.1038/ncomms2290 PMC3535359

[192] Zhang X, Jiang Q, Shen Y. et al. Using landscape genomics to assess local adaptation of fruit trees to current and future climatic conditions. Fruit Res. 2024a;4:0

[193] Cheng B, Du W, Bourke PM. et al. Population genetics of horticultural crops aided by multi-omics technology and its implications for ornamental plants. Ornam Plant Res. 2024;4:0

[194] Zhang Q, Zhang H, Sun L. et al. The genetic architecture of floral traits in the woody plant *Prunus mume*. Nat Commun. 2018;9:1702 29703940 10.1038/s41467-018-04093-z PMC5923208

[195] Zheng T, Li P, Zhuo X. et al. The chromosome-level genome provides insight into the molecular mechanism underlying the tortuous-branch phenotype of *Prunus mume*. New Phytol. 2022;235:141–56 34861048 10.1111/nph.17894 PMC9299681

[196] Feng Y, Sun Q, Zhang G. et al. Genome-wide identification and characterization of ABC transporters in nine *Rosaceae* species identifying *MdABCG28* as a possible cytokinin transporter linked to dwarfing. Int J Mol Sci. 2019;20:5783 31744249 10.3390/ijms20225783 PMC6887749

[197] Ge Y, Lai Q, Luo P. et al. Transcriptome profiling of *Gerbera hybrida* reveals that stem bending is caused by water stress and regulation of abscisic acid. BMC Genomics. 2019;20:600 31331262 10.1186/s12864-019-5961-1 PMC6647082

[198] Yadav LK, Wilde HD. Identification and bioinformatic characterization of rare variants of *Rhododendron canescen*s architecture genes. Euphytica. 2022;218:66

[199] Chen S, Zhang Y, Zhang T. et al. Comparative transcriptomic, anatomical and phytohormone analyses provide new insights into hormone-mediated tetraploid dwarfing in hybrid sweetgum (*Liquidambar styraciflua* x *L. formosana*). Front Plant Sci. 2022;13:924044 35832220 10.3389/fpls.2022.924044 PMC9271929

[200] Qin S, Fu S, Yang Y. et al. Comparative microscopic, transcriptome and IAA content analyses reveal the stem growth variations in two cultivars *Ilex verticillata*. Plants (Basel). 2023;12:1941 37653858 10.3390/plants12101941 PMC10220661

[201] Ju Y, Feng L, Wu J. et al. Transcriptome analysis of the genes regulating phytohormone and cellular patterning in *lagerstroemia* plant architecture. Sci Rep. 2018;8:15162 30310123 10.1038/s41598-018-33506-8 PMC6181930

[202] Yu C, Hou K, Zhang H. et al. Integrated mass spectrometry imaging and single-cell transcriptome atlas strategies provide novel insights into taxoid biosynthesis and transport in *Taxus mairei* stems. Plant J. 2023a;115:1243–60 37219365 10.1111/tpj.16315

[203] Li J, Sheng Y, Xu H. et al. Transcriptome and hormone metabolome reveal the mechanism of stem bending in water lily (*Nymphaea tetragona*) cut-flowers. Front Plant Sci. 2023;14:1195389 37746018 10.3389/fpls.2023.1195389 PMC10515221

[204] Nobori T, Oliva M, Lister R. et al. Multiplexed single-cell 3D spatial gene expression analysis in plant tissue using PHYTOMap. Nat Plants. 2023;9:1026–33 37308583 10.1038/s41477-023-01439-4 PMC10356616

[205] Tung CC, Kuo SC, Yang CL. et al. Single-cell transcriptomics unveils xylem cell development and evolution. Genome Biol. 2023;24:3 36624504 10.1186/s13059-022-02845-1 PMC9830878

[206] Zhuo X, Zheng T, Li S. et al. Identification of the *PmWEEP* locus controlling weeping traits in *Prunus mume* through an integrated genome-wide association study and quantitative trait locus mapping. Hortic Res. 2021;8:131 34059642 10.1038/s41438-021-00573-4 PMC8167129

[207] Hollender CA, Pascal T, Tabb A. et al. Loss of a highly conserved sterile alpha motif domain gene (*WEEP*) results in pendulous branch growth in peach trees. Proc Natl Acad Sci USA. 2018;115:E4690–9 29712856 10.1073/pnas.1704515115 PMC5960274

[208] Kohler AR, Scheil A, Hill JL Jr. et al. Defying gravity: WEEP promotes negative gravitropism in peach trees by establishing asymmetric auxin gradients. Plant Physiol. 2024;195:1229–55 38366651 10.1093/plphys/kiae085 PMC11142379

[209] Khodakovskaya M, Vankova R, Malbeck J. et al. Enhancement of flowering and branching phenotype in chrysanthemum by expression of ipt under the control of a 0.821 kb fragment of the *LEACO1* gene promoter. Plant Cell Rep. 2009;28:1351–62 19533142 10.1007/s00299-009-0735-x

[210] Yin J, Chang X, Kasuga T. et al. A basic helix-loop-helix transcription factor, PhFBH4, regulates flower senescence by modulating ethylene biosynthesis pathway in petunia. Hortic Res. 2015;2:15059 26715989 10.1038/hortres.2015.59 PMC4680862

[211] Xie Q, Chen G, Chen X. et al. Jointly silencing *BoDWARF*, *BoGA20ox* and *BoSP* (*SELF-PRUNING*) produces a novel miniature ornamental *Brassica oleracea* var. *acephala* f. tricolor variety. Mol Breed. 2014;34:99–113

[212] Liang YC, Reid MS, Jiang CZ. Controlling plant architecture by manipulation of gibberellic acid signalling in petunia. Hortic Res. 2014;1:14061 26504556 10.1038/hortres.2014.61 PMC4596332

[213] Tang Y, Lu L, Sheng Z. et al. An R2R3-MYB network modulates stem strength by regulating lignin biosynthesis and secondary cell wall thickening in herbaceous peony. Plant J. 2023c;113:1237–58 36633057 10.1111/tpj.16107

[214] Zhao W, Ding L, Liu J. et al. Regulation of lignin biosynthesis by an atypical bHLH protein CmHLB in *chrysanthemum*. J Exp Bot. 2022b;73:2403–19 35090011 10.1093/jxb/erac015

[215] Gargul JM, Mibus H, Serek M. Manipulation of *MKS1* gene expression affects *Kalanchoe blossfeldiana* and *Petunia* hybrida phenotypes. Plant Biotechnol J. 2015;13:51–61 25082411 10.1111/pbi.12234

[216] Nakagawa H, Jiang CJ, Sakakibara H. et al. Overexpression of a petunia zinc-finger gene alters cytokinin metabolism and plant forms. Plant J. 2005;41:512–23 15686516 10.1111/j.1365-313X.2004.02316.x

[217] Snowden KC, Simkin AJ, Janssen BJ. et al. The decreased apical dominance1/Petunia hybrida CAROTENOID CLEAVAGE DIOXYGENASE8 gene affects branch production and plays a role in leaf senescence, root growth, and flower development. Plant Cell. 2005;17:746–59 15705953 10.1105/tpc.104.027714 PMC1069696

[218] Yamagishi M, Nomizu T, Nakatsuka T. Overexpression of lily MicroRNA156-resistant SPL13A stimulates stem elongation and flowering in *Lilium formosanum* under non-inductive (non-chilling) conditions. Front Plant Sci. 2024;15:1456183 39494055 10.3389/fpls.2024.1456183 PMC11527630

[219] Zheng R, Peng Y, Chen J. et al. The genome-level survey of the WOX gene family in *Melastoma dodecandrum* Lour. Int J Mol Sci. 2023;24:17349 38139178 10.3390/ijms242417349 PMC10743900

[220] Tang Y, Lu L, Huang X. et al. The herbaceous peony transcription factor WRKY41a promotes secondary cell wall thickening to enhance stem strength. Plant Physiol. 2023b;191:428–45 36305685 10.1093/plphys/kiac507 PMC9806655

[221] Suh E-J, Hong J, k., Lee, Y.-H., Kim, D. C. Overexpression of the *Brassica rapa* SRS7 gene in pot-type chrysanthemum [*Chrysanthemum morifolium* Ramat] reduces plant height. Sci Hortic. 2020;273:109634

[222] Suh E-J, Kim DC, Park SR. et al. Development of a compact garden mum variety through introduction of the *BrSRS7* gene. Sci Hortic. 2023;309:111589

[223] Lütken H, Jensen LS, Topp SH. et al. Production of compact plants by overexpression of *AtSHI* in the ornamental Kalanchoë. Plant Biotechnol J. 2010;8:211–22 20051037 10.1111/j.1467-7652.2009.00478.x

[224] Duan H, Wu B, Qin H. et al. Harnessing *agrobacterium rhizogenes* and mobile elements for innovative transgene-free gene editing in woody plants. Ornam Plant Res. 2025;5:e027

[225] Shvets DY, Berezhneva ZA, Musin KG. et al. Rol genes of agrobacteria: possible biological functions. Biol Bull Rev. 2023;13:S359–76

[226] Casanova E, Trillas MI, Moysset L. et al. Influence of rol genes in floriculture. Biotechnol Adv. 2005;23:3–39 15610964 10.1016/j.biotechadv.2004.06.002

[227] Pellegrineschi A, Damon J-P, Valtorta N. et al. Improvement of ornamental characters and fragrance production in lemon-scented geranium through genetic transformation by *agrobacterium rhizogenes*. Bio/Technology. 1994;12:64–8

[228] Christensen B, Sriskandarajah S, Serek M. et al. Transformation of *Kalanchoe blossfeldiana* with rol-genes is useful in molecular breeding towards compact growth. Plant Cell Rep. 2008;27:1485–95 18597094 10.1007/s00299-008-0575-0

[229] Favero BT, Tan Y, Lin Y. et al. Transgenic *Kalanchoe blossfeldiana*, containing individual rol genes and open reading frames under 35S promoter, exhibit compact habit, reduced plant growth, and altered ethylene tolerance in flowers. Front Plant Sci. 2021;12:672023 34025708 10.3389/fpls.2021.672023 PMC8138453

[230] Cheng H, Zhang F, Ding Y. CRISPR/Cas9 delivery system engineering for genome editing in therapeutic applications. Pharmaceutics. 2021;13:1649 34683943 10.3390/pharmaceutics13101649 PMC8538656

[231] Li Y, Li W, Li J. The CRISPR/Cas9 revolution continues: from base editing to prime editing in plant science. J Genet Genomics. 2021;48:661–70 34362681 10.1016/j.jgg.2021.05.001

[232] Saini H, Thakur R, Gill R. et al. CRISPR/Cas9-gene editing approaches in plant breeding. GM Crops Food. 2023;14:1–17 10.1080/21645698.2023.2256930 PMC10512805 37725519

[233] Piovesan A, Vancauwenberghe V, Van De Looverbosch. et al. X-ray computed tomography for 3D plant imaging. Trends Plant Sci. 2021;26:1171–85 34404587 10.1016/j.tplants.2021.07.010

[234] Zhang Y, Wang J, Du J. et al. Dissecting the phenotypic components and genetic architecture of maize stem vascular bundles using high-throughput phenotypic analysis. Plant Biotechnol J. 2021c;19:35–50 32569428 10.1111/pbi.13437 PMC7769239

[235] Hu Z, Liu J, Shen S. et al. Large-volume fully automated cell reconstruction generates a cell atlas of plant tissues. Plant Cell. 2024b;36:4840–61 39283506 10.1093/plcell/koae250 PMC11852339

[236] Bai B, Yang X, Li Y. et al. Deep learning-enabled virtual histological staining of biological samples. Light Sci Appl. 2023;12:57 36864032 10.1038/s41377-023-01104-7 PMC9981740

[237] Latonen L, Koivukoski S, Khan U. et al. Virtual staining for histology by deep learning. Trends Biotechnol. 2024;42:1177–91 38480025 10.1016/j.tibtech.2024.02.009

[238] Zhang B, Gao Y, Zhang L. et al. The plant cell wall: biosynthesis, construction, and functions. J Integr Plant Biol. 2021a;63:251–72 33325153 10.1111/jipb.13055

[239] Colares CJG, Pastore TCM, Coradin VTR. et al. Near infrared hyperspectral imaging and MCR-ALS applied for mapping chemical composition of the wood specie *Swietenia Macrophylla* king (mahogany) at microscopic level. Microchem J. 2016;124:356–63

[240] Li Y, Shen W, Zhang X. et al. Single-cell characterization of major components of plant cell walls in situ by Raman spectroscopy. Sci China Life Sci. 2024c;67:1772–4 38644446 10.1007/s11427-024-2542-4

[241] Wallace JG, Rodgers-Melnick E, Buckler ES. On the road to breeding 4.0: unraveling the good, the bad, and the boring of crop quantitative genomics. Annu Rev Genet. 2018;52:421–44 30285496 10.1146/annurev-genet-120116-024846

[242] Yu H, Bai S, Li J. Towards breeding 5.0: smart variety by intelligent breeding. Chin Sci Bull. 2024;69:4687–90

[243] Tang J, Ye J, Liu P. et al. Ornamental plant gene editing: past, present and future. Ornam Plant Res. 2023a;3:1–6

[244] Din A, Wani MA, Jin C. et al. Post-genomic era of CRISPR/Cas technology in ornamental plants: advantages, limitations, and prospects. Ornam Plant Res. 2025;5:0

